# Biological Evaluation, DFT Calculations and Molecular Docking Studies on the Antidepressant and Cytotoxicity Activities of *Cycas pectinata* Buch.-Ham. Compounds

**DOI:** 10.3390/ph13090232

**Published:** 2020-09-03

**Authors:** Jinnat Rahman, Abu Montakim Tareq, Md. Mohotasin Hossain, Shahenur Alam Sakib, Mohammad Nazmul Islam, Md. Hazrat Ali, A. B. M. Neshar Uddin, Muminul Hoque, Mst. Samima Nasrin, Talha Bin Emran, Raffaele Capasso, A. S. M. Ali Reza, Jesus Simal-Gandara

**Affiliations:** 1 Department of Pharmacy, International Islamic University Chittagong, Kumira, Chittagong 4318, Bangladesh; jinnatrahman14@gmail.com (J.R.); montakim0.abu@gmail.com (A.M.T.); mdjisan16@gmail.com (M.M.H.); sayeadiiuc@gmail.com (M.N.I.); hazratpharm@gmail.com (M.H.A.); nesharuddinemon1@gmail.com (A.B.M.N.U.); muminul359@gmail.com (M.H.); shathy_ru@yahoo.com (M.S.N.); 2 Department of Theoretical and Computational Chemistry, University of Dhaka, Dhaka 1000, Bangladesh; sakibhasaniiuc@gmail.com; 3 Department of Pharmacy, BGC Trust University Bangladesh, Chittagong 4381, Bangladesh; talhabmb@bgctub.ac.bd; 4 Department of Agricultural Sciences, University of Naples Federico II, 80055 Portici, Italy; 5 Department of Biochemistry and Molecular Biology, University of Chittagong, Chittagong 4331, Bangladesh; 6 Nutrition and Bromatology Group, Department of Analytical and Food Chemistry, Faculty of Food Science and Technology, University of Vigo—Ourense Campus, E32004 Ourense, Spain

**Keywords:** *Cycas pectinata*, MAO, serotonin, antidepressant, cytotoxicity, oximes, molecular docking, DFT calculations, cyclopentadecanone, oxime

## Abstract

*Cycas pectinata* Buch.-Ham. is commonly used in folk medicine against various disorders. The present study investigated the antidepressant and cytotoxicity activity of methanol extract of *C. pectinata* (MECP) along with quantitative phytochemical analysis by GC-MS method. Here, the GC-MS study of MECP presented 41 compounds, among which most were fatty acids, esters, terpenoids and oximes. The antidepressant activity was assessed by the forced swimming test (FST) and tail suspension test (TST) models. In contrast, MECP (200 and 400 mg/kg) exhibited a significant and dose-dependent manner reduction in immobility comparable with fluoxetine (10 mg/kg) and phenelzine (20 mg/kg). MECP showed a weak toxicity level in the brine shrimp lethality bioassay (ED_50_: 358.65 µg/mL) comparable to the standard drug vincristine sulfate (ED_50_: 2.39 µg/mL). Three compounds from the GC-MS study were subjected to density functional theory (DFT) calculations, where only cyclopentadecanone oxime showed positive and negative active binding sites. Cyclopentadecanone oxime also showed a good binding interaction in suppressing depression disorders by blocking monoamine oxidase and serotonin receptors with better pharmacokinetic and toxicological properties. Overall, the MECP exhibited a significant antidepressant activity with moderate toxicity, which required further advance studies to identify the mechanism.

## 1. Introduction

Depression is a condition characterized by a lowering of the mood and dislike for movement that may distress an individual’s thoughts, conduct, emotions, and comfort [1]. Depressive behavior is additionally connected with suicide, which ranges from 10 and 20 million each year [2,3]. According to the World Health Organization (WHO) report, around 450 million people have a mental disorder, which may rise to 15% by 2020 [4]. In addition, the physical changes additionally happen in extreme, vital, or melancholia or melancholic depression. These comprise sleep deprivation or hypersomnia, modified eating disorders, anorexia and weight reduction and several endocrine dysfunctions with alterations in body temperature. Depressive behavior is the feature of some psychiatric disorders, which may also be caused by somewhat normal life situations; for example, deprivation of sleep, sicknesses, or an adverse effect of drugs and clinical treatments. Patients with major depressive behavior have several symptoms that may reflect in the brain, monoamine synapses or neurotransmitters, explicitly norepinephrine, serotonin, and dopamine [5,6,7].

There are several antidepressant drugs available to treat depression, but the rate of success of first-line therapy for depression [e.g., selective serotonin reuptake inhibitors (SSRIs) and serotonin-norepinephrine reuptake inhibitors (SNRIs)] is low due to several limitations (adverse effects, lower response and the onset of action, etc.), which have been mentioned in several reviews [8]. Thus, it is imperative that new antidepressant drugs demonstrate improvement of these drawbacks. Several phytochemicals (alkaloids, flavonoids, sterol, terpenes) were reported to have an antidepressant effect [9]. Oximes (R_1_R_2_C = NOH) are chemicals containing nitrogen produced by organisms in all kingdoms of life [10]. In recent years, oxime derivatives were reported to have several pharmacological activities: cytotoxicity, antibiotic effect, anticonvulsant, antimicrobial, cardiac dysrhythmia, antinociceptive activities [11,12,13,14].

Presently monoamine oxidase A (MAO-A) is useful in the treatment of depression disorders, because MAO metabolizes serotonin or 5-hydroxytryptamine (5-HT) in the central nervous system (CNS) [15]. SSRIs are effective in depression, but due to their limitations, the evaluation of new bioactive substances is a major target for the researchers [16]. Oxime derivatives are reported to have an antidepressant effect [17,18,19], whereas chalcone oxime ethers are reported to have potent inhibitory activity against MAO-B [20]. Our present study design aimed at the evaluation of the biological activity along with a computational study (DFT, molecular docking, ADME/T), where the MAO-A and serotonin receptor are used as a molecular targets for oxime derivatives in depression disorders.

*Cycas pectinata* Buch.-Ham. (Family: Cycadaceae), commonly known as moniraj or nagmoni, belongs to the genus Cycas [21]. This plant has traditionally been useful for hair growth, curing stomach aches, and curing ulcers [22,23]. Various ethnopharmacological uses in different treatment aspects are documented for Cycas species. *Cycas revoluta* Thunb. was used for inflammation, vomiting and tonic conditions [24], while *Cycas circinalis* L. is used for healing wounds and swollen glands. *Cycas rumphii* Miq. male pollen and cones are reported to have strong narcotic effects [25]. Like in a previous *C. pectinata* study, a number of fatty acid methyl esters along with other compounds have been reported for *C. revolute* [26], whereas 16 different bioactive compounds have been reported for *C. circinalis* [27]. In our previous study, several secondary metabolites from the methanol extract of *C. pectinata* exhibited the following pharmacological activities, including antioxidant, anti-inflammatory, thrombolytic, anxiolytic, sedative, antinociceptive and antidiarrheal properties [22]. In the present study we report the antidepressant activity along with the cytotoxicity activity of *C. pectinate* to find a potential lead compound from *C. pectinata* in alleviating depression disorders by blocking monoamine oxidase (MAOs) and serotonin receptors. To explain this possible mechanism of action of compounds isolated from *C. pectinata*, we also performed a quantum chemical analysis (DFT calculations) with molecular docking, and ADME/T studies to reveal the potential target(s) for inhibition of the human MAO and serotonin receptors.

## 2. Results and Discussion

### 2.1. Qualitative and Quantitative Phytochemical Analysis with Acute Toxicity Study

Phytochemical analysis is useful to evaluate the therapeutic and physiological activities of a plant extract. A qualitative phytochemical screen is performed to determine the presence or absence of secondary plant metabolites. The investigation showed positive results for carbohydrates, alkaloids, phenol, proteins, flavonoids, and saponins (data not shown), which was similar to our previous study that reported similar results [22]. The phytochemical analysis of *C. pectinata* leaves showed the presence of several phytochemicals. Glycosides are a group of compounds with drug-likeness and numerous studies have suggested that they are a fruitful source of potential drugs. Flavonoids are reported to have anti-inflammatory and anti-cancer activity, whereas tannins possess anti-inflammatory and anti-microbial activity [28]. Phenolic compounds are also present, which possess various physiological functions like anti-aging, anti-inflammation, anti-apoptosis, anti-carcinogenic, inhibition of angiogenesis and enhancement of endothelial function [29].

A total of 66 compounds were identified in the GC-MS analysis, whereas 25 compounds were reported by Tareq et al. [22]. In addition, 41 other compounds are presented in Table 1 and Figure S1, most of which were esters, organic compounds and alcohols. The most abundant compounds along with their retention times were (E)-2-decen-1-ol (20.360), chloroacetic acid 4-pentadecyl ester (20.360), glycerol 1-palmitate (20.009), octadecanoic acid 2-hydroxy-1,3-propanediyl ester (20.009), hexadecanoic acid 2-hydroxy-1-(hydroxymethyl)ethyl ester (20.009), docosanoic acid docosyl ester (19.440), cyclopentadecanone oxime (19.440), and 1-O-(16-hydroxyhexadecyl)-d-mannitol (19.440). These compounds isolated from MECP could help develop a new drug for depression and cancer diseases. The antidepressant activity was evaluated in Swiss albino mice, which required a prior toxicity study. Before starting the experiments and the acute toxicity study of MECP at 400–2000 mg/kg dose was conducted in Swiss albino mice. The methanol extract of *C. pectinata* leaves was determined to be safe. There was no change of behavioral rush or mortality, morbidity in 8 h observation period of 400, 600, 800, 1000, 2000 mg/kg of MECP doses which were similar to the previous study [22].

### 2.2. Antidepressant Activity

Anxiety and depression are mental conditions that may recur and are generally undiagnosed and untreated. Physical problems might join these mental conditions, and patients frequently present in medical care centers with physical problems instead of mental situations or problems [30]. Though several antidepressant drugs available, but the rate of success is falling day by day (e.g., SSRIs and SNRIs) [8,31]. Thus, the phytochemical study is a topic of interest for the researcher to evaluate a lead compound to treat depression. Several phytochemicals (alkaloids, flavonoids, sterol, terpenes) are reported to have antidepressant effects [9]. Additionally, a few medicinal plants such as *M. angolensis* [32], *N. sativa* [33] *R. rosea* [34] are reported to have bilateral anxiolytic and antidepressant effects. In our previous study, MECP showed decreased locomotor activity with a significant anxiolytic activity and also a strong binding affinity against the human serotonin receptor (PDV: 5I6X) suggested by the interacted compounds [22]. Here, the antidepressant activity of MECP was evaluated by a tail suspension test (TST) and forced swimming test (FST), which are the most promising models to assess antidepressant activity.

Moreover, TST is proposed to have a higher pharmacological sensitivity as compared to FST. In these models, the immobility time or the period of time when animals stopped struggling was calculated, where the antidepressant agents reduce the immobility time in rodents [35]. Fluoxetine is a SSRI very widely used as an antidepressant agent, which basically works by inhibiting access of serotonin transporter protein into presynaptic serotonin neurons by inhibiting the transporter protein and also has mild activity at the 5-hydroxytryptamine 2A (5HT2A) and 5-hydroxytryptamine 2C (5HT2C) receptors [36]. Also, phenelzine was used in this study is a monoamine oxidase inhibitor (MAOI) that acts by inhibiting MAO activity and afterward raises the neural concentration of neurotransmitters, thus increasing monoamine secretion in the synaptic cleft and alleviating depression [37].

**Figure 1:**
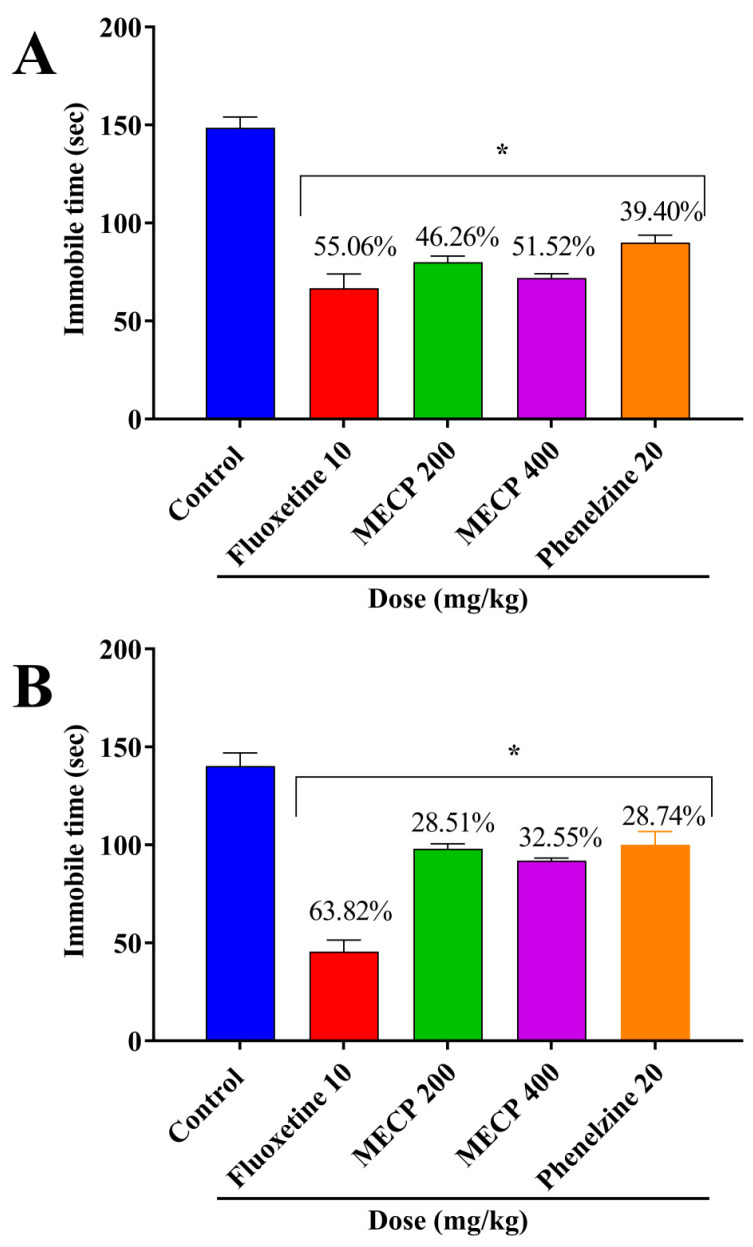
Antidepressant effect of methanol extract of *C. pectinata* leaves (MECP), fluoxetine and phenelzine in tail suspension test (**A**) and forced swimming test (**B**). The values are shown as mean ± standard error of the mean (SEM). * *p* < 0.001 statistically significant compared with the control by Dunnett’s test (*n* = 5).

In our study, both doses of MECP showed significant antidepressant activity, leading to a possibility that MECP may act in the presynaptic serotonin neurons by inhibiting serotonin transporter protein and by inhibiting the activity of MAO. In TST, 200 and 400 mg/kg dose exhibited 46.26% and 51.52% inhibition in immobility, whereas the standard drugs fluoxetine and phenelzine exhibited 55.06% and 39.40%, respectively. Additionally, the FST showed significant (*p* < 0.001) immobility, whereas the 200 and 400 mg/kg exhibited 28.51% and 32.55% inhibition of immobility. The results are presented in Figure 1. The presence of alkaloids and saponins in MECP may be a possible reason for this antidepressant activity as well as the presence of oxime derivatives [17,18,19,38].

### 2.3. Cytotoxicity Activity

**Figure 2:**
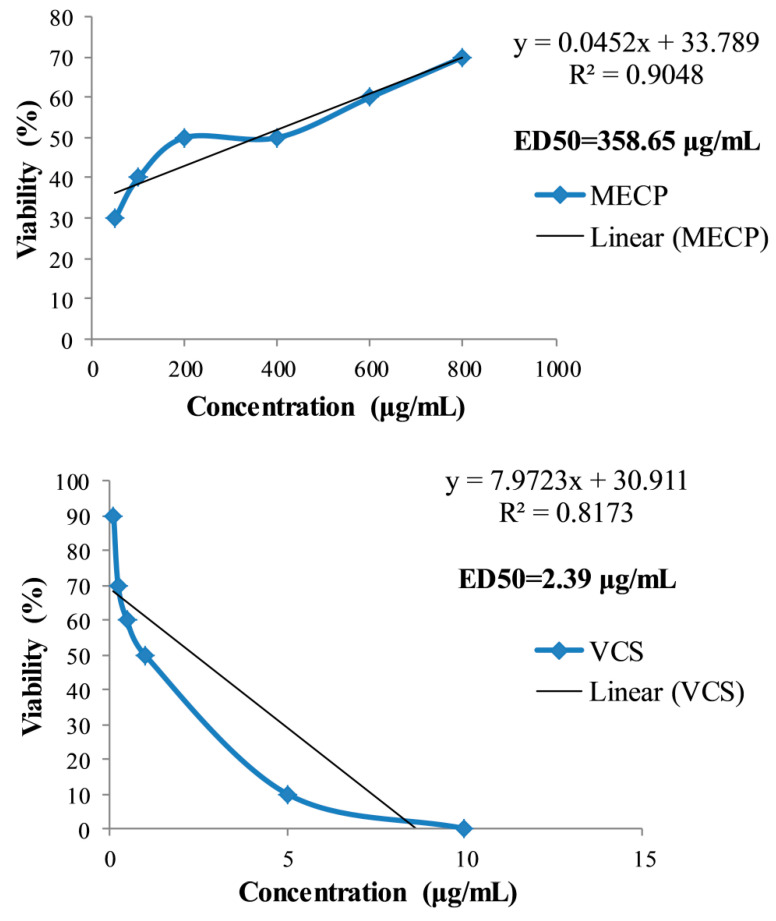
Percentage of mortality of brine shrimp lethality bioassay of methanol extract of *C. pectinata* leaves (MECP) and standard drug vincristine sulfate (VCS) at different concentrations.

The evaluation of the bioactivity of plant products by the brine shrimp lethality bioassay is an effective, safe and economical method. A good correlation is found in the brine shrimp lethality bioassay with solid human tumors for cytotoxic and pesticidal activity, which is useful for the discovery of active antitumor agents and natural pesticides [39]. This method is also used as a pre-screening test for antitumor research. Generally, the higher the ED_50_, the lower the toxicity of the extract is and vice versa [40]. In our study, the ED_50_ of the test samples was calculated using a concentration against the viability of the nauplii. Vincristine sulfate demonstrated the viability of nauplii when the concentration gradually decreased from 10 μg/mL (zero viability) to 0.125 μg/mL (90% viability). MECP has an ED_50_ of 358.65 µg/mL, which is weakly toxic, whereas the standard drug vincristine sulfate exhibited 2.39 µg/mL (highly toxic). The results are presented in Figure 2. This moderate toxicity level of MECP may be due to the presence of oxime derivatives, which reported to have cytotoxicity and antitumor activity [11,41].

### 2.4. In Silico Study

#### 2.4.1. Molecular Geometry

**Figure 3:**
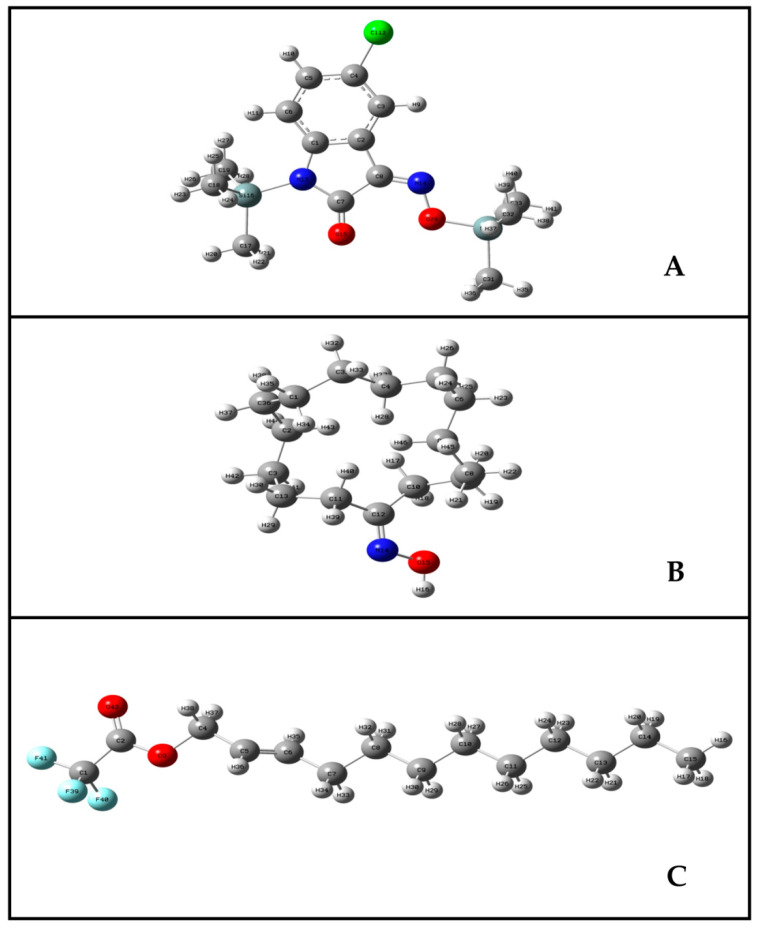
Optimized geometric structures of 5-chloro-1-(trimethylsilyl)-1H-indole-2,3-dione 3-[O-(trimethylsilyl)oxime] (**A**); cyclopentadecanone oxime (**B**) and *trans*-2-dodecen-1-ol trifluoroacetate (**C**).

The stable configurations of 5-chloro-1-(trimethylsilyl)-1*H*-indole-2,3-dione 3-[O-(trimethylsilyl)oxime], cyclopentadecanone oxime; and *trans*-2-dodecen-1-ol trifluoroacetate obtained from the conformational analysis which has been used for reactivity analysis are shown in Figure 3 with the numbering of atoms. From the structural point of view, these three compounds belongs to the C1 point group symmetry group and hence all the calculated frequencies transform to the same A symmetry species. 

The total energies of the three compounds calculated by the B3LYP method are −1845.68068, 718.77081 and −997.04879 Hartree, respectively (Table 2). Among the three compounds *trans*-2-dodecen-1-ol trifluoroacetate showed a higher dipole moment value. Dipole moments tell us about the charge separation in a molecule. The larger the difference in electronegativity of bonded atoms, the larger the dipole moment [42]. Among the three isolated compound, 5-chloro-1-(trimethylsilyl)-1*H*-indole-2,3-dione 3-[O-(trimethylsilyl)oxime] has higher polarizability value. Generally, polarizability increases as the volume occupied by electrons increases. In atoms, this occurs because larger atoms have more loosely held electrons than smaller atoms with tightly bound electrons [43].

#### 2.4.2. Charges and MESP Calculations

The atomic charges (Mulliken and NBO) play an important role in molecular polarizability, dipole moment, electronic structure, molecular reactivity and a lot of related properties of molecular systems. The charge distributions over the atoms suggest the formation of donor and acceptor pairs involving the charge transferring the molecule. The charges on the atoms of the present 5-chloro-1-(trimethylsilyl)-1*H*-indole-2,3-dione 3-[O-(trimethylsilyl)oxime]; cyclopentadecanone oxime and *trans*-2-dodecen-1-ol trifluoroacetate; were calculated by Mulliken population analysis [44] and NBO charges [45] using B3LYP method with 6-31G+ (d,p) basis set, the tabular representation of the results are presented in Tables S1–S3.

For 5-chloro-1-(trimethylsilyl)-1*H*-indole-2,3-dione 3-[O-(trimethylsilyl)oxime] it can be easily seen that the highest positive Mulliken charge value of 1.710 a.u was accommodated on the Si_16_ atom that is attached to the pyrrole ring, while in NBO charges the highest positive value was 1.905 a.u on the Si_30_ atom which connect with O atom. Also, the Mulliken charge with the highest negative value of (−0.862~−0.877) a.u was on the methyl group C atom wherein NBO charges provide the highest negative value of (−1.219~−1.225) a.u on the methyl group C atom. Due to the electron-withdrawing nature of the methyl group, its C atom is pulling electrons towards it. 

As for cyclopentadecanone oxime, it showed the highest positive Mulliken charge value of 0.428 a.u accommodated on the H_16_ atom, which is bonded to the O atom. The highest negative Mulliken charge value of −0.638 a.u belongs to the O_15_ atom which is attached to the N atom. The natural atomic charges value is in excellent agreement with the highest positive and negative Mulliken charge values for the same atom of the molecule. From the table it can be easily seen regarding the Mulliken charge values for *trans*-2-dodecen-1-ol trifluoroacetate, the highest positive value of 0.769 a.u was accommodated on the C_1_ atom which is bond with CF_3_. This natural atomic charge value also agreed with the obtained result for the same carbon. It shows the highest positive value was 0.983 a.u. In Mulliken charges, the highest negative value of −0.480 a.u is accommodated on the O_3_ atom. This natural atomic charge value does not also agree with the obtained result for the same atom, whereas it shows the highest negative value of −0.524 a.u is accommodated on O_42_ atom at molecule. From the charges calculation the highest positive and negative value of Mulliken and NBO charge of atoms sometimes did not agreed with each other due to the two methods used.

**Figure 4:**
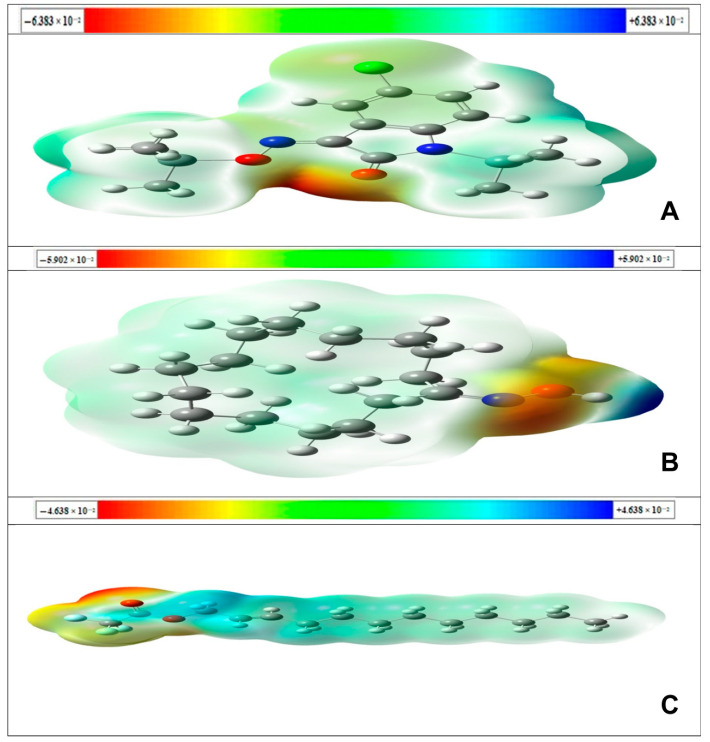
Calculated 3D surface mapped of electrostatic potential for 5-chloro-1-(trimethylsilyl)-1*H*-indole-2,3-dione 3-[O-(trimethylsilyl)oxime] (**A**); cyclopentadecanone oxime (**B**); *trans*-2-dodecen-1-ol trifluoroacetate (**C**), respectively in (a.u), the electron density isosurface being 0.0004 (a.u).

The molecular electrostatic potential (MESP) surface [46] from Figure 4 illustrates the molecules’ charge distributions three-dimensionally. This map allows us to visualize variably charged regions of a molecule. The knowledge of the charge distributions can be used to determine how molecules may interact with one another and it is also used to determine the nature of their chemical bonds [47]. The MESP map was checked out by theoretical calculations using the B3LYP/6-31G+ (d,p) level. Molecular electrostatic potential shows the electronic density and is useful in recognizing sites for electrophilic attack, nucleophilic reactions, and hydrogen bonding interactions. Different colors represent the different values of the electrostatic potential at the surface. The negative areas (red, orange and yellow color) of MESP were related to electrophilic reactivity, the positive areas (blue color) ones to nucleophilic reactivity and green color are neutral regions. This figure also provides a visual representation of the chemically active sites and the comparative reactivity of atoms. The computed 3D plot of MESP for the title compounds is depicted in the figure, based on the electron density at different points on the molecule. However, potential values of the three isolated compounds ranges from −6.383 × 10^−2^ a.u (deepest red) to +6.383 × 10^−2^ a.u (deepest blue), −5.902 × 10^−2^ a.u (deepest orange) to +5.902 × 10^−2^ a.u (deepest blue), −4.638 × 10^−2^ a.u (deepest red) to +4.638 × 10^−2^ a.u (deepest blue) respectively.

According to the MESP map in the figure for 5-chloro-1-(trimethylsilyl)-1*H*-indole-2,3-dione 3-[O-(trimethylsilyl)oxime], cyclopentadecanone oxime and *trans*-2-dodecen-1-ol trifluoroacetate; the negative regions are associated with the O_15_, O_15_, and O_42_ atoms, respectively. Therefore these atom positions are suitable sites for electrophilic attack. Alternatively, only cyclopentadecanone oxime showed a positive region associated with the H_16_ atom that indicates that this atom can be the most probably involved in nucleophilic processes. Here, 5-chloro-1-(trimethylsilyl)-1*H*-indole-2,3-dione 3-[O-(trimethylsilyl)oxime]; and *trans*-2-dodecen-1-ol trifluoroacetate; didn’t show any nucleophilic attack sites. The presence of positive and negative binding sites in cyclopentadecanone oxime may result in good interactions with proteins in biological systems.

#### 2.4.3. FMOs and Global Descriptors

**Figure 5:**
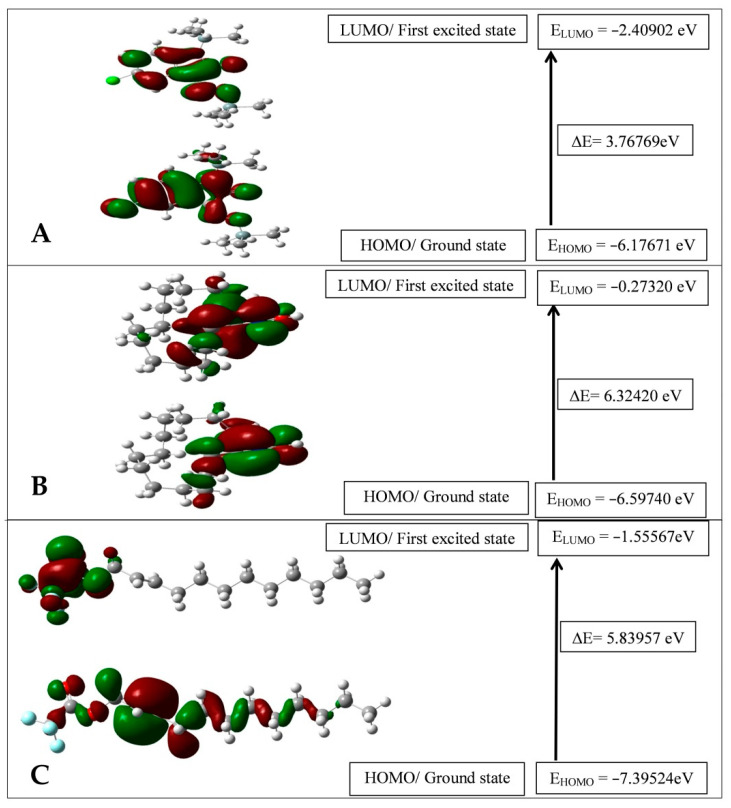
HOMO-LUMO plot 5-chloro-1-(trimethylsilyl)-1*H*-indole-2,3-dione 3-[O-(trimethylsilyl)-oxime] (**A**); cyclopentadecanone oxime (**B**); trans-2-dodecen-1-ol trifluoroacetate (**C**), respectively, by B3LYP/6-31G+ (d,p) level of theory.

The frontier molecular orbitals, HOMO and LUMO, are the most important orbitals in a molecule. They play an important role in the optical and electric properties, as well as in quantum chemistry and the UV–Vis spectra [48]. The highest occupied molecular orbital (HOMO), represents the ionization potential of the molecule and lowest occupied molecular orbital (LUMO), corresponding electron affinity value is called the frontier molecular orbitals (FMOs) showed in Figure 5 were calculated at the B3LYP/6-31G+ (d,p) level for the three isolated compounds. 

These orbitals determine the way how the molecule interacts with other species and give information about the reactivity/stability of specific regions of the molecule. The energy of HOMO characterizes the electron-donating ability of a molecule, while LUMO energy determines the ability to accept an electron. Therefore, higher values of E_HOMO_ indicate a better tendency towards the donation of an electron. From Figure 5, *trans*-2-dodecen-1-ol trifluoroacetate is the better molecule which has the ability to accept electrons while the energy value of HOMO (E_HOMO_ = −7.39524 eV) that allows it to be the best electron donor molecule. The energy gap between the HOMO and LUMO is very important in determining a molecule’s chemical reactivity. A high value of the energy gap indicates that the molecule shows high chemical stability; indicates a hard molecule, while a small HOMO-LUMO gap means small excitation energies to the manifold of excited states, and action as a soft molecule. Among three isolated compounds, 5-chloro-1-(trimethylsilyl)-1*H*-indole-2,3-dione 3-[O-(trimethylsilyl)oxime] shows the lowest energy gap indicating it is more reactive than the two other compounds.

Using Koopmans’ theorem [49,50] (I) and (A) values can be correlated with the frontier orbitals by the relation: I = −E_HOMO_ and A = −E_LUMO_. Ionization potential (I) is defined as the amount of energy needed to remove an electron from a molecule. High ionization energy indicates high stability, chemical inertness and small ionization energy indicating high reactivity of the atoms and molecules. Trans-2-Dodecen-1-ol, trifluoroacetate has the lowest ionization potential value (I = 7.39524 eV), which indicates that it is the best electron donor. The electronic affinity (A) is defined as the energy released when an electron is added to a neutral molecule. A molecule with high (A) values tends to accept electrons easily. From Table 3 it is clear that 5-chloro-1-(trimethylsilyl)-1*H*-*I*ndole-2,3-dione 3-[O-(trimethylsilyl)oxime] is the most reactive. The global chemical reactivity descriptors such as chemical potential (µ), electronegativity (χ), hardness (η), softness (S), and electrophilicity index (ω) which were calculated from the HOMO and LUMO energies were obtained at the level of theory B3LYP/6-31G+ (d,p) and are incorporated in Table 3. 

According to these parameters, the chemical reactivity varies with the structural configuration of the molecules. Global reactivity descriptors such as chemical potential denote as (µ = -χ), the absolute electronegativity (χ) is given by the relation (χ = (IP + EA)/2), global hardness and global softness (S) are defined as (η = (E_LUMO_ − E_HOMO_)/2) and (S = 1/2η), the electrophilicity (ω) can be calculated using the electronic chemical potential and the chemical hardness (ω = µ^2^/2η) [51,52,53,54,55]. Hardness (η) and softness (S) are useful concepts for understanding the behavior of chemical systems. A hard molecule has a large energy gap and a soft molecule has a small energy gap [56]. Therefore, soft molecules will be more polarizable than hard molecules. From the established theoretical calculations cyclopentadecanone oxime has the highest hardness value (η = 3.16210 eV), which indicates that it is the hardest molecule. 5-Chloro-1-(trimethylsilyl)-1*H*-indole-2,3-dione 3-[O-(trimethylsilyl)oxime] has the highest softness (S = 0.53083eV), so it is the softest molecule. The chemical potential µ (eV) measures the escaping tendency of an electron and it can be associated with the molecular electronegativity [57] then, as µ becomes more negative, it is more difficult to lose an electron but easier to gain one. As shown in Table 3, *trans*-2-dodecen-1-ol trifluoroacetate is the least stable and the most reactive among all the compounds. Electronegativity (χ), represents the ability of molecules to attract electrons. The (χ) values displayed in Table 3 show that cyclopentadecanone oxime; has the higher electronegativity (4.47546 eV) value compared to all the other molecules. Electrophilicity (ω), that gives an idea of the stabilization energy when the system gets saturated by electrons, which come from the external environment. This reactivity information shows if a molecule is capable of donating charges. A good, more reactive nucleophile is characterized by a lower value of (ω), while higher values indicate the presence of a good electrophile. Our results indicate that cyclopentadecanone oxime has lower values of (ω), so that compound is a good nucleophile, whereas *trans*-2-dodecen-1-ol, trifluoroacetate is a good electrophile.

#### 2.4.4. Vibrational Spectral Analysis

The vibrational band assignments had been performed based on the normal coordinate analysis. Internal coordinates of three isolated compounds had constructed according to Pulay’s recommendations [58]. The calculated wavenumbers were selectively scaled according to the scaled quantum mechanical (SQM) method recommended by Rauhut and Pulay [59] using a scale factor with the root mean square (RMS) wavenumber error, which is in the reasonable limit for proper assignment. The observed FT-IR and simulated theoretical spectra calculated at the B3LYP/6-31G+ (d, p) basis set are shown in Figure S2. The calculated wavenumbers and their assignments are also presented in Table 4. The detailed analyses of vibrational wavenumbers for various functional groups are discussed below.

#### 2.4.5. Hydroxyl (O–H) Group Vibrations

Bands due to O–H stretching are of medium to strong intensity in the infrared spectrum, although it may be broad. For solids, liquids and concentrated solutions a broad band of less intensity is normally observed [60]. The very weak FT-IR band at 3696 cm^−1^ is assigned to the O–H stretching vibrations. Normally free O–H stretching vibrations appeared around 3600 cm^−1^ for phenols [61]. The observed broad intense IR band for cyclopentadecanone oxime at corresponds to O–H stretching mode, which is calculated at 3884 cm^−1^.

#### 2.4.6. C-H Vibrations

Aromatic compounds commonly exhibit multiple weak bands in the 3100–3000 cm^−1^ region due to aromatic C-H stretching vibrations and also in-plane bending vibrations generally lie in the range 1000–1300 cm^−1^ [62]. The bands appearing at (3081 ~ 3099) cm^−1^, (3100 ~ 3200) cm^−1^, (3023 ~ 3069) cm^−1^ for 5-chloro-1-(trimethylsilyl)-1*H*-indole-2,3-dione 3-[O-(trimethylsilyl)oxime], cyclopentadecanone oxime and *trans*-2-dodecen-1-ol trifluoroacetate; respectively in the FT-IR spectrum are assigned to C–H ring stretching vibrations. In the present study, the C–H in-plane bending vibrations of 5-chloro-1-(trimethylsilyl)-1*H*-indole-2,3-dione 3-[O-(trimethylsilyl)oxime] and *trans*-2-dodecen-1-ol trifluoro-acetate is identified at 1137 cm^−1^ and 989 cm^−1^ at the B3LYP methods are assigned to C–H in-plane bending vibrations.

#### 2.4.7. Methylene (H-C-H) group Vibrations 

Methyl groups are generally referred to as electron donating substituents in an aromatic ring system. Whenever a methyl group is present in a compound, it gives rise to asymmetric and symmetric stretching vibrations [63]. The asymmetric stretch is usually at a higher wavenumber than the symmetric stretch. The asymmetric stretching vibrations of CH_3_ are expected in the 2925–3000 cm^−1^ region and the symmetric CH_3_ stretching vibrations in the 2905–2940 cm^−1^ range [64,65]. The predicted asymmetric and symmetric stretching vibrations for CH_3_ are at (2927~3017) cm^−1^, (3051~3093) cm^−1^, (2920~3001) cm^−1^ and (2916 ~ 2919) cm^−1^, (3007~3019) cm^−1^, (2805~2917) cm^−1^ for 5-chloro-1-(trimethylsilyl)-1*H*-indole-2,3-dione 3-[O-(trimethylsilyl)oxime], cyclopentadecanone oxime and *trans*-2-dodecen-1-ol trifluoroacetate, respectively. Furthermore, the observed peaks at (1464~1459) cm^−1^, (1262 cm^−1^~1267) cm^−1^, 1237 cm^−1^ can be assigned to the scissoring, twisting and wagging modes of CH_3_ and CH_2_ groups in aliphatic chains, respectively [66]. The predicted scissoring, wagging and twisting vibrations for CH_2_ are at (1459~1499) cm^−1^, (1346~1373) cm^−1^, (1246~1287) cm^−1^ and (1407~1506) cm^−1^, (1324~1373) cm^−1^, (1287~1312) cm^−1 1^ for cyclopentadecanone oxime; and *trans*-2-dodecen-1-ol trifluoroacetate, respectively.

#### 2.4.8. C-N Vibrations

The identification of C = N vibrations is a difficult task since mixing of vibrations is possible in this region. Silverstein et al. [67] assigned the C = N stretching absorption in the 1690–1640 cm^−1^ range for aromatic amines. The present work shows that the theoretically computed value of C = N stretching vibrations band observed at 1593 cm^−1^ and 1759 cm^−1^ in the FT-IR spectrum for 5-chloro-1-(trimethylsilyl)-1*H*-indole-2,3-dione 3-[O-(trimethylsilyl)oxime] and cyclopentadecanone oxime, respectively.

#### 2.4.9. C=C Vibrations

The phenyl ring CC stretching vibrations are generally observed between 1600–1400 cm^−1^ [68], in which the bands between 1600–1500 cm^−1^ are assigned to C=C stretching and the rest to C-C stretching, even though no such distinction is present within the ring. In the present study, the bands observed at (1555~1579) cm^−1^ and (1680~1689) cm^−1^ are assigned to C=C for 5-chloro-1-(trimethylsilyl)-1*H*-indole-2,3-dione 3-[O-(trimethylsilyl)oxime] and *trans*-2-dodecen-1-ol trifluoroacetate, respectively.

#### 2.4.10. Carbonyl (C=O) Group Vibration

The C=O stretching vibrations give rise to the characteristic bands in IR spectra, and the intensity of these bands can increase owing to the conjugation or formation of hydrogen bonds. The C=O stretching of ketones is expected in the region 1760–1730 cm^−1^ [69]. C=O stretching mode is not an independent vibrational mode because of it coupled with the vibrations of adjacent groups. The FT-IR band with strong intensity at 1717 cm^−1^ alone was assigned to the carbonyl stretching mode of 5-chloro-1-(trimethylsilyl)-1*H*-indole-2,3-dione 3-[O-(trimethylsilyl)oxime]. 

#### 2.4.11. NMR Analysis

After the optimization of molecular geometry of the three isolated compounds the ^1^H and ^13^C nuclear magnetic resonance (NMR) chemical shift values calculated at the B3LYP/6-31G+ (d,p) level in chloroform solvents by comparing their observed values in CDCl_3_ solvent with respect to TMS as an internal reference [70]. The theoretically calculated ^1^H- and ^13^C-NMR chemical shift values are presented in Table 5 and Table 6. The theoretically determined ^1^H-and ^13^C-NMR spectra are shown in Figures S3 and S4, respectively.

The ^1^H atoms chemical shift values of 1H-Indole-2,3-dione, 5-chloro-1-(trimethylsilyl)-, 3-[O-(trimethylsilyl)oxime] are divided into two ranges; the first range is approximately 0~6.5 ppm, the second range is around 0~−0.956 ppm. The first group is due to the H atoms in the benzene ring and methyl group. The second group is due to the H atoms in the methyl group attached to Si atoms. Also, ^1^H atoms chemical shift values of cyclopentadecanone, oxime divide into two ranges; the first one is around 0~4.5 ppm, the second one is greater than 4.5 ppm. The first group is due to the H atoms in the cyclic alkyl chain and that atoms have slightly positive charges. The highest chemical shift was found for H_16_ atom which associated with the O atom. Lastly, the chemical shift values of ^1^H atoms for trans-2-Dodecen-1-ol, trifluoroacetate are divided into two ranges; the first one is around 0~5 ppm, the second one is greater than 5 ppm. Frist range chemical shift values determined those H atoms in the alkyl chain and showed a slightly positive charge. The highest chemical shifts were found for H_35_, H_36_ atoms which associated with the C atom nearly O atom. Due to different chemical atmospheres at various regions around the H atoms for 1H-Indole-2,3-dione, 5-chloro-1-(trimethylsilyl)-, 3-[O-(trimethylsilyl)oxime]; cyclopentadecanone, oxime; and trans-2-Dodecen-1-ol, trifluoroacetate the chemical shift inequality had originated.

The calculated ^13^C chemical shift values of for 5-chloro-1-(trimethylsilyl)-1*H*-indole-2,3-dione 3-[O-(trimethylsilyl)oxime] are in the −15~143 ppm range. This range is divided into two parts; the first range is greater than 100 ppm for C_7_, C_8_, C_1_, C_4_, C_5_, C_2_, C_3_ atoms which are located in the benzene ring. The second range is less than 100 ppm for C_6_, C_17_, C_33_, C_19_, C_18_, C_32_, C_31_ atoms that alkyl chain carbons attached to silicon atoms. Also, the ^13^C chemical shift values of cyclopentadecanone oxime are in the 11~147 ppm range. This range divided into two part; firstly 146.55 ppm was found for O bonded C_12_ atom and below 100 ppm corresponds to the C_11_, C_10_, C_8_, C_36_, C_1_, C_13_, C_3,_ C_4_, C_31_, C_5_, C_6_, C_7_, C_2_, C_9_ atoms in the cyclic alkyl chain. Finally, the *trans*-2-dodecen-1-ol trifluoroacetate chemical shift values are found in the 3~152 ppm range. The highest chemical shift values were found at 151.74, 129.32, 121.59 and 105.65 ppm for the C_2_, C_6_, C_1_, and C_5_ atoms that are bonded with a highly negative charge O atom. Besides, less than 100 ppm values are found for the C_4_, C_7_, C_13_, C_12_, C_11_, C_10_, C_9,_ C_8_, C_14_ and C_15_ atoms that are located in the straight alkyl chain.

#### 2.4.12. Molecular Docking Study

Computer-aided drug design (CADD) plays a significant role in developing new drugs. There are mainly two types of drug design methods available, namely: structure-based and ligand-based drug design [71]. In our previous study, we used ligand-based interactions to select a lead compound with sedative activity, which exhibited a significant binding affinity towards the human serotonin receptor (PDB: 5I6X) [22]. Here, the MAO receptor is used because MAO-A is generally targeted to treat depression and anxiety, whereas MAO-B useful for Alzheimer’s disease (AD) and Parkinson’s disease [72]. As oxime derivatives were reported to have antidepressant effects [17,18,19], in our present study, human monoamine oxidase A (PDB: 2Z5X) was used for a molecular docking study of antidepressant activity. The antidepressant activity is presented in Table 7. In the present study, cyclopentadecanone oxime and *trans*-2-dodecen-1-ol trifluoroacetate showed the highest and lowermost binding affinity against human monoamine oxidase A (PDB: 2Z5X), with docking scores of −4.333 kcal/mol and −3.155 kcal/mol, respectively. 5-Chloro-1-(trimethylsilyl)-1*H*-indole-2,3-dione 3-[O-(trimethylsilyl)oxime] did not show any interaction, whereas the standard drug phenelzine showed −5.324 kcal/mol binding affinity. Cyclopentadecanone oxime interacted with the monoamine oxidase A (PDB: 2Z5X) by one π-alkyl interaction to Phe 112. The interaction of the compounds is presented in Figure S5.

Human serotonin receptor (PDB: 5I6X) used also for the molecular docking study, where cyclopentadecanone oxime and *trans*-2-dodecen-1-ol trifluoroacetate exhibited the highest and lowermost binding affinity, with docking scores of −6.537 kcal/mol and −2.387 kcal/mol, respectively The standard drug fluoxetine shows a −9.07 kcal/mol interaction. Cyclopentadecanone oxime interacted with the human serotonin receptor (PDB: 5I6X) by one H-bond to Asp 98 and one alkyl interaction to Ile 172. The interaction of the compounds is presented in Figure S6.

The molecular docking study of cytotoxicity activity was performed against the human estrogen receptor (PDB ID: 1ERR) and epidermal growth factor receptor tyrosine kinase (PDB ID: 1M17). Cyclopentadecanone oxime gave a −7.685 kcal/mol and −4.59 kcal/mol binding interaction against the human estrogen receptor (PDB ID: 1ERR) and epidermal growth factor receptor tyrosine kinase (PDB ID: 1M17), whereas the standard drug vincristine sulfate exhibited −3.896 kcal/mol and −3.85 kcal/mol interactions, respectively. Cyclopentadecanone oxime interacted with the human estrogen receptor (PDB: 1ERR) through one H-bond to Glu 353, one π-alkyl interaction to Phe 404 and an alkyl-interaction to Leu 346. In addition cyclopentadecanone oxime interacted with the epidermal growth factor receptor tyrosine kinase (PDB ID: 1M17) through one H-bond to Met 769, and two alkyl-interactions to Val 702 and Leu 820. The interactions of the compounds are presented in Figures S7 and S8.

#### 2.4.13. ADME/T and Toxicological Properties Analysis

ADME properties and drug toxicity are important in preventing the early introduction of drugs into the commercial market. From a business point of view, it is necessary to remove the poor pharmacokinetic profile compounds, which reduces the cost of the drug development stage. As a result, over the previous decade, ADME/T screening has been applied in the early drug discovery phase [73]. The selected isolated compounds from the methanol extract of *C. pectinata* were subjected to the ADME/T profiling by following Lipinski’s (Rule of Five) [74] and Veber’s rules [75]. The three compounds 5-chloro-1-(trimethylsilyl)-1*H*-indole-2,3-dione 3-[O-(trimethylsilyl) oxime]; cyclopentadecanone oxime; and *trans*-2-dodecen-1-ol trifluoroacetate satisfy Lipinski’s Rule of Five, whereas *trans*-2-dodecen-1-ol trifluoroacetate violated Veber’s rules (Table 8). 

In the toxicological study, 5-chloro-1-(trimethylsilyl)-1*H*-indole-2,3-dione 3-[O-(trimethylsilyl)-oxime] did not exhibit any risk of toxicity, whereas cyclopentadecanone oxime and *trans*-2-dodecen-1-ol trifluoroacetate showed risks of Ames toxicity and carcinogenic effect, respectively (Table 9). Aside from these effects, all three compounds can be considered as lead compounds with antidepressant and cytotoxicity activity.

## 3. Materials and Methods

### 3.1. Chemicals

Fluoxetine (Square Pharmaceuticals Ltd., Dhaka, Bangladesh), phenelzine (Ranbaxy Laboratories, Haryana, India), Tween-80 (Sigma Aldrich Co., St. Louis, MO, USA), and vincristine sulfate (2 mg/vial) (Beacon Pharmaceuticals Ltd. Dhaka, Bangladesh) were purchased from a local trader. All other chemicals were analytical grade.

### 3.2. Plant Materials and Preparation of Crude Extract 

The details of the *C. pectinata* leaves (MECP) plant material were described in our earlier study [22]. The freshly collected leaves were ground into a coarse powder using a grinder (NOWAKE, Hokuto, Japan). The maceration of powder and methanol solvent was followed in a 1:4 ratio, with filtration by Whatman filter paper (#1) after seven days. The filtration was followed by evaporation in a water bath (40 °C) to obtain a crude extract. The crude extract was kept under refrigeration at 4 °C until further use.

### 3.3. Experimental Animals

The average weight of 25–35 g of six-seven weeks old Swiss albino mice of both sexes was obtained from the animal house of Department of Pharmacy, International Islamic University of Chittagong (IIUC), Chittagong, Bangladesh. The animals were adapted with the laboratory condition (room temperature 25 ± 2 °C, relative humidity 55–60%) by supplying food pellets and water. For the use of the experiment, all the animals were adapted for 14 days with laboratory conditions. The study was approved by the Institutional Animal Ethical Committee, Department of Pharmacy, International Islamic University Chittagong, Bangladesh, according to governmental guidelines under the reference (Pharm/p&d/138/13-′19,22/12/2019) [76].

### 3.4. GC-MS (Gas Chromatography-Mass Spectroscopy) Analysis of MECP

The detailed gas chromatography-mass spectroscopy (GC-MS) analysis of the methanol extract of *C. pectinata* leaves (MECP) were described in the earlier study of Tareq et al. [22].

### 3.5. Acute Toxicity Study

The acute oral toxicity of methanol extract of *C. pectinata* was determined by the OECD (2002) guidelines No. 423 method [77]. Mice were divided into six groups, where each group contained five animals. The first group received 1% Tween-80 in normal saline. The other groups were received 400, 600, 800, 1000, 2000 mg/kg of MECP dose. Then all the animals were observed for 8 h to detect early symptoms such as behavioral changes or mortality, morbidity and later for 3 days.

### 3.6. Phytochemical Screening

In the preliminary phytochemical screening of freshly prepared methanol leaves crude extract was qualitatively tested for the determination of carbohydrates, alkaloids, glycosides, tannins, terpenoids, flavonoids, and saponins [78,79].

### 3.7. Antidepressant Activity

#### 3.7.1. Experimental Design for Anti-Depressant Activity

The antidepressant activity of the extract evaluated by the tail suspension test and forced swimming test. The mice were divided into four groups (*n* = 5). Administration of extract/control to the animals was followed after 60 min prior to study [80,81]:

Group I: Negative control received 1% Tween-80 (10 mL/kg, b.w.) orally
Group II: Positive control phenelzine received 20 mg/kg b.w. I.P.
Group III: Positive control fluoxetine received 10 mg/kg b.w. I.P.
Group IV: Received MECP 200 mg/kg b.w. orally
Group V: Received MECP 400 mg/kg b.w. orally

#### 3.7.2. Tail Suspension Test (TST)

The antidepressant activity of MECP was executed by the method described by Steru et al. [80]. The treatment was followed as described in Section 3.7.1. After 60 min of treatment, each mouse was suspended by using adhesive tape at the tip of the tail over the rim of a box. Then the immobility time was recorded from the 6 min suspended period, whereas the first 2 min for initial adjustment and last 4 min for immobility time:

$$\mathrm{Inhibition}\;\left(\%\right)=\frac{A-B}{A}\;\times 100$$

where, A = immobile time in the control group; B = immobile time in the test group.

#### 3.7.3. Forced Swimming Test (FST)

The antidepressant activity of MECP was evaluated by the forced swimming test, as described by Porsolt et al., [81]. A glass box (25 × 15 × 25 cm^3^) filled to 15 cm with water (25 ± 2 °C) was utilized as a test apparatus for swimming. The treatment was followed as described in Section 3.7.1. After 60 min of treatment, each mouse was forced to swim in the apparatus. The immobility time was calculated from the 6 min swimming period. When the mice stopped struggling and remained suspended in water was considered as the immobility time and the period is recorded.

$$\mathrm{Inhibition}\;\left(\%\right)=\frac{A-B}{A}\;\times 100$$

where, A = immobile time in the control group; B = immobile time in the test group.

### 3.8. Brine Shrimp Lethality Bioassay

The brine shrimp lethality bioassay was followed to evaluate the cytotoxicity of methanol extract of *C. Pectinata* leaves by Meyer et al. [39]. In 1000 mL distilled water, 38 g NaCl was dissolved to prepare artificial seawater. NaOH was added to maintain the pH at 8.0. Then serially diluted concentrations of 50, 100, 200, 400, 600 and 800 μg/mL were obtained. Vincristine sulfate used as a positive control as the preceding method in a serial concentration dilution 0.125, 0.25, 0.5, 1, 5 and 10 μg/mL. Then ten matured live shrimp were placed in all test tubes at room temperature (25 ± 1 °C) and after 24 h, each test tube was assessed, and the number of alive nauplii was counted and recorded.

% of viability = (N_l_/N_0_) ×100

where, N_0_ = Number of nauplii taken; N_l_ = Number of nauplii alive

### 3.9. In Silico Study

#### 3.9.1. Quantum Chemical Analysis

Quantum chemical analysis was performed with the Gaussian 09 software package [82] via the Gauss view 6.0.10 [83] molecular visualization program on a Pentium IV/3.02Hz personal computer. The selected isolated compounds 5-chloro-1-(trimethylsilyl)-1*H*-indole-2,3-dione 3-[O-(trimethylsilyl) oxime], cyclopentadecanone oxime and *trans*-2-dodecen-1-ol trifluoroacetate were fully optimized at the level of density functional theory (DFT) using the B3LYP with the 6-31G+ (d,p) basis set. The minima of the potential energy hypersurfaces were considered to be the stationary points and confirmed from the absence of any imaginary frequency. Electronic properties, such as HOMO-LUMO energies, molecular electrostatic potential (MESP) were calculated using the B3LYP method, based on the optimized structure in the gas phase. Furthermore, Mulliken and natural bond orbital (NBO) charges and global reactivity descriptors of the proposed compounds were analyzed. Calculated vibrational frequencies were multiplied by a suitable scaling number (0.964) [84] to better match experimental frequencies. Besides, the ^1^H and ^13^C nuclear magnetic resonance (NMR) chemical shift [85] (with respect to a TMS reference and chloroform solution) of the proposed compounds were also carried out by GIAO method in same method and level of basis set.

#### 3.9.2. Molecular Docking Study

The optimized structure of 5-chloro-1-(trimethylsilyl)-1*H*-indole-2,3-dione 3-[O-(trimethylsilyl) oxime], cyclopentadecanone oxime; and *trans*-2-dodecen-1-ol trifluoroacetate were subjected to a molecular docking study according to Sastry et al. as briefly explained in Adnan et al. [86,87]. The proteins used for the docking study were retrieved from the Protein data bank (https://www.rcsb.org/structure/): human monoamine oxidase A (PDB ID: 2Z5X), human serotonin (PDB ID: 5I6X), human estrogen receptor (PDB ID: 1ERR), and epidermal growth factor receptor tyrosine kinase (PDB ID: 1M17) [88]. The molecular docking study was performed using Schrödinger (Maestro v11.1).

#### 3.9.3. ADME/T and Toxicological Properties Analysis

The optimized structures of 5-chloro-1-(trimethylsilyl)-1*H*-Indole-2,3-dione 3-[O-(trimethylsilyl) oxime], cyclopentadecanone oxime and *trans*-2-dodecen-1-ol trifluoroacetate were subjected to ADME/T following the rules of Lipinski (Rule of Five) [74] and Veber [75]. In addition, the toxicological properties were analyzed by the admetSAR (http://lmmd.ecust.edu.cn/admetsar2/). The ADME/T analysis was evaluated by SwissADME (http://www.swissadme.ch/) [89].

### 3.10. Statistical Analysis

The values are shown as mean ± standard error mean (SEM). * *p* < 0.001 statistical significance was calculated by one-way ANOVA (Dunnett’s test) using the GraphPad Prism (version 8.4.) software (San Diego, CA, USA).

## 4. Conclusions

This study reports that methanol leaves extract of *C. pectinata* could be a potential source of compounds with antidepressant and cytotoxicity activity due to the presence of secondary metabolites. In addition, the computational study of the oxime derivatives by DFT and molecular docking study unveiled better binding interaction against the MAO and serotonin receptor with good pharmacokinetic and toxicological properties. Further advanced studies are recommended to identify the mechanism of action of *C. pectinata*.

## Figures and Tables

**Table 1:** Quantitative compounds identified from methanol extract of *C. pectinata* by GC-MS analysis.

Sl. No.	RT	Compound Name	*m/z*	Area	PA (%)	Molecular Formula	MW (g/mol)	Class
1	5.881	1*H*-Indole-2,3-dione, 5-chloro-1-(trimethylsilyl)-, 3-[O-(trimethylsilyl)oxime]	73.00	851549	3.80	C_14_H_21_ClN_2_O_2_Si_2_	340.95	Oxime
2	11.640	3-Octyn-2-ol	44.00	25927	0.12	C_8_H_14_O	126.2	Fatty alcohol
3	11.640	2-Cyclohexen-1-one, 3-(3-hydroxybutyl)-2,4,4-trimethyl-	44.00	25927	0.12	C_13_H_22_O_2_	210.31	Ketone
4	11.640	Bioallethrin	44.00	25927	0.12	C_19_H_26_O_3_	302.4	Pyrethroid
5	11.640	3-Nonyn-2-ol	44.00	25927	0.12	C_9_H_16_O	140.22	Secondary alcohol
6	12.516	1-Octadecyne	43.00	209536	0.94	C_18_H_34_	250.5	Hydrocarbon
7	12.516	*Z*-2-Dodecenol	43.00	209536	0.94	C_12_H_24_O	184.32	Fatty alcohol
8	12.516	Phytol, acetate	43.00	209536	0.94	C_22_H_42_O_2_	338.6	Diterpene
9	12.515	5-Nonadecen-1-ol	81.00	122608	0.55	C_19_H_38_O	282.5	Alcohols
10	12.515	2-Tridecyne	81.00	122608	0.55	C_13_H_24_	180.33	Alkyne
11	12.516	9-Eicosyne	43.00	187141	0.84	C_20_H_38_	278.5	Alkyne
12	12.516	Dodecanal	43.00	187141	0.84	CH_3_(CH_2_)_10_CHO	184.32	Aldehyde
13	12.516	*trans*-2-Dodecen-1-ol, trifluoroacetate	43.00	187141	0.84	C_14_H_23_F_3_O_2_	280.33	Ester
14	13.450	Tridecanoic acid, 12-methyl-, methyl ester	74.00	417474	1.86	C_15_H_30_O_2_	242.4	Fatty acid
15	13.450	Eicosanoic acid, methyl ester	74.00	417474	1.86	C_21_H_42_O_2_	326.6	FAME
16	13.450	Octadecanoic acid, 17-methyl-, methyl ester	74.00	417474	1.86	C_20_H_40_O_2_	312.5	FAME
17	15.170	13-Tetradece-11-yn-1-ol	67.00	47905	0.21	C_14_H_24_O	208.34	Alcohol
18	15.170	9,12-Octadecadienoic acid, methyl ester, (*E,E*)-	67.00	47905	0.21	C_19_H_34_O_2_	294.5	FAME
19	15.339	Cyclopropaneoctanoic acid, 2-[[2-[(2-ethyl- cyclopropyl)methyl]cyclopropyl]methyl]-, methyl ester	55.00	70317	0.31	C_22_H_38_O_2_	334.5	Fatty acid
20	15.339	3-Tetradecyn-1-ol	55.00	70317	0.31	C_14_H_26_O	210.36	Alkyne
21	15.339	7-Hexadecenoic acid, methyl ester, (*Z*)-	55.00	70317	0.31	C_17_H_32_O_2_	268.4	Fatty acid
22	15.339	Ethyl iso-allocholate	55.00	70317	0.31	C_26_H_44_O_5_	436.6	Steroid
23	15.337	Isophytol, acetate	71.00	172950	0.77	C_22_H_42_O_2_	338.6	Diterpene
24	15.337	*E*-2-Tetradecen-1-ol	71.00	172950	0.77	C_14_H_28_O	212.37	Alkyne
25	15.478	Tetradecanoic acid, 12-methyl-, methyl ester, (*S*)-	74.00	107333	0.96	C_16_H_32_O_2_	316.5	FAME
26	15.478	Heptacosanoic acid, methyl ester	74.00	107333	0.48	C_28_H_56_O_2_	424.7	Fatty acid
27	15.478	Cyclopentanetridecanoic acid, methyl ester	74.00	107333	0.48	C_19_H_36_O_2_	296.5	Fatty acid
28	16.199	Dodecanoic acid, 2-(acetyloxy)-1-[(acetyloxy)methyl]ethyl ester	73.00	111054	0.49	C_19_H_34_O_6_	338.5	Ester
29	16.199 and 5.881	Phloroglucitol	73.00	111054 and 851549	0.49 and 3.80	C_6_H_12_O_3_	132.16	Alcohol
30	16.604	Octadecanal, 2-bromo-	44.00	11355	0.05	C_18_H_35_BrO	347.4	Aldehyde
31	16.604 and 12.515	Undecanal	81.00 and 44.00	122608 and 11355	0.05 and 0.55	C_10_H_21_CHO	170.29	Aldehyde
32	17.623	Octasiloxane, 1,1,3,3,5,5,7,7,9,9,11,11,13,13,15,15-hexadecamethyl-	73.00	77448	0.35	C_16_H_50_O_7_Si_8_	577.2	Volatile organic compound
33	17.623	Dodecanoic acid, 2,3-bis(acetyloxy)propyl ester	73.00	77448	0.35	C_19_H_34_O_6_	358.5	Ester
34	19.440	D-Mannitol, 1-O-(16-hydroxyhexadecyl)-	73.00	77215	0.34	C_22_H_46_O_7_	422.6	Alcohol
35	19.440	Cyclopentadecanone, oxime	73.00	77215	0.34	C_15_H_29_NO	239.4	Oxime
36	19.440	Docosanoic acid, docosyl ester	73.00	77215	0.34	C_44_H_88_O_2_	649.2	Emollient
37	20.009	Hexadecanoic acid, 2-hydroxy-1-(hydroxymethyl)ethyl ester	44.00	49605	0.22	C_19_H_38_O_4_	330.5	Fatty acid glycerol ester
38	20.009	Octadecanoic acid, 2-hydroxy-1,3-propanediyl ester	44.00	49605	0.22	C_39_H_76_O_5_	625.0	Monoalkyl ester
39	20.009	Glycerol 1-palmitate	44.00	49605	0.22	C_19_H_38_O_4_	330.5	Fatty acid
40	20.360	Chloroacetic acid, 4-pentadecyl ester	44.00	24894	0.11	C_17_H_33_ClO_2_	304.9	Ester
41	20.360	2-Decen-1-ol, (*E*)-	44.00	24894	0.11	C_10_H_20_O	156.26	Fatty acid

RT: Retention Time; *m/z*: m stands for mass and z stands for the charge number of ions, PA: Peak Area, MW: Molecular weight; FAME: fatty acid methyl ester.

**Table 2:** Optimized energies of 5-chloro-1-(trimethylsilyl)-1*H*-indole-2,3-dione 3-[O-(trimethylsilyl)-oxime]; cyclopentadecanone oxime and *trans*-2-dodecen-1-ol trifluoroacetate with dipole moment and polarizability.

Compounds	Energy (a.u)	Dipole Moment (Debye)	Polarizability (a.u)
5-Chloro-1-(trimethylsilyl)-1*H*-indole-2,3-dione 3-[O-(trimethylsilyl)oxime]	−1845.68068	1.367	261.403
Cyclopentadecanone oxime	−718.77081	0.712	162.046
*trans*-2-Dodecen-1-ol trifluoroacetate	−997.04879	4.968	159.682

**Table 3:** Global reactivity descriptors values in the gas phase.

Global Reactivity Descriptors	5-Chloro-1-(trimethylsilyl)-1*H*-indole-2,3-dione 3-[O-(trimethyl-silyl)oxime]	Cyclopentadecanone Oxime	*trans*-2-Dodecen-1-ol Trifluoroacetate
Ionisation potential (I) eV	6.17671	6.59740	7.39524
Electron affinity (A) eV	2.40902	0.27320	1.55567
Chemical hardness (η)	1.88385	3.16210	2.91979
Softness (S)	0.53083	0.31625	0.34249
Chemical potential (μ)	−4.29287	−3.43530	−4.47546
Electronegativity (χ)	4.29287	3.43530	4.47546
Electrophilicity index (ώ)	9.21434	5.90064	10.01485

**Table 4:** Calculated scaled infra-red (IR) frequencies (cm^−1^) for 5-chloro-1-(trimethylsilyl)-1*H*-indole-2,3-dione 3-[O-(trimethylsilyl)oxime], cyclopentadecanone oxime and *trans*-2-dodecen-1-ol trifluoroacetate, respectively by DFT B3LYP/6-31+G (d,p) method (atom positions numbered as in the table).

MD	5-Chloro-1-(trimethyl-silyl)-1*H*-indole-2,3-dione 3-[O-(trimethylsilyl)oxime]	MD	Cyclopentadecanone Oxime	MD	*trans*-2-Dodecen-1-ol Trifluoroacetate
-	-	υ(O_15_-H_16_)	3884	-	-
υ(C- H)	3081~3099	υ(C- H)	3100~3200	υ(C- H)	3023~3069
υ_Asy_(H-C-H)	2927~3017	υ_Asy_(H-C-H)	3051~3093	υ_Asy_(H-C-H)	2920~3001
υ_Sy_(H-C-H)	2916~2919	υ_Sy_(H-C-H)	3007~3019	υ_Sy_(H-C-H)	2805~2917
-	-	δ_S_(H-C-H)	3023 ~ 3037	-	-
υ(C=C)_Aro_	1555~1579	-	-	υ(C=C)	1680~1689
υ(C_4_=Cl_12_)	695	-	-	υ(C-C)	1283
-	-	δ_S_(H-C-H)	1459~1499	δ_S_(H-C-H)	1407~1506
-	-	δ_W_(H-C-H)	1346~1373	δ_W_(H-C-H)	1324~1373
-	-	δ_T_(H-C-H)	1246~1287	δ_T_(H-C-H)	1287~1312
δ_W_(C-H)_Aro_	916	-	-	δ(C-H)	989
δ(C-H)_Aro_	1137	-	-	-	-
υ(C_7_=O_15_)	1717	-	-	-	-
υ(C_18_=N_14_)	1593	υ(C_12_=N_14_)	1759	-	-
υ(O_29_-N_14_)	1035/979	υ(O_15_-N_14_)	891	-	-
-	-	δ_S_(C_12_-N_14_-O_15_)	524	-	-
-	-	-	-	υ(C-F)	1123~1169

Calculated values were corrected by multiplying the frequency factor, f = 0.964. MD = Mode of Vibration, υ = Stretching, υ_Sy_ = Symmetric Stretching, υ_Asy_ = Asymmetric Stretching, δ = Bending, δ_S_ = Scissoring, δ_W_ = Wagging, δ_R_ = Rocking, δ_T_ = Twisting, F^S^ = Scaled frequency, Aro = Aromatic.

**Table 5:** Calculated ^1^H-NMR isotropic chemical shift (TMS and chloroform solution) by the DFT/B3LYP/6-31G+ (d,p) method (atom positions are numbered in the table).

	Compound (Chemical Shift-ppm)				
Proton No.	5-Chloro-1-(trimethylsilyl)- 1*H*-indole-2,3-dione 3-[O- (trimethylsilyl)oxime]	Proton No.	Cyclopentadecanone Oxime	Proton No.	*trans*-2-Dodecen-1-ol, Trifluoroacetate
9-H	6.364	16-H	4.643	35-H	5.567
10-H	6.129	18-H	2.074	36-H	5.183
11-H	5.943	40-H	1.809	38-H	4.266
22-H	0.097	17-H	1.221	37-H	3.952
21-H	0.078	39-H	1.126	33-H	1.459
27-H	−0.276	28-H	1.119	34-H	1.319
25-H	−0.281	19-H	1.068	31-H	0.699
39-H	−0.364	43-H	0.971	30-H	0.645
34-H	−0.387	46-H	0.736	19-H	0.611
40-H	−0.412	30-H	0.705	20-H	0.607
36-H	−0.420	24-H	0.673	28-H	0.600
24-H	−0.621	27-H	0.637	24-H	0.596
28-H	−0.630	20-H	0.587	26-H	0.594
42-H	−0.743	25-H	0.574	23-H	0.593
37-H	−0.759	34-H	0.570	27-H	0.592
23-H	−0.848	29-H	0.524	22-H	0.582
26-H	−0.851	37-H	0.520	29-H	0.579
35-H	−0.904	41-H	0.508	21-H	0.579
20-H	−0.918	33-H	0.505	25-H	0.579
41-H	−0.948	38-H	0.505	32-H	0.503
38-H	−0.956	21-H	0.478	16-H	0.245
		35-H	0.395	18-H	0.131
		22-H	0.354	17-H	0.124
		44-H	0.316		
		42-H	0.298		
		23-H	0.256		
		32-H	0.252		
		45-H	0.248		
		26-H	0.181		

**Table 6:** Calculated ^13^C-NMR isotropic chemical shift (TMS and chloroform solution) by the DFT/B3LYP/6-31G+ (d,p) method (atom positions numbered in the table).

	Compound (Chemical Shift-ppm)				
Carbon No.	5-Chloro-1-(trimethylsilyl)-1*H*-indole-2,3-dione 3-[O-(trimethylsilyl)oxime]	Carbon No.	Cyclopentadecanone Oxime	Carbon No.	*trans*-2-Dodecen-1-ol Trifluoroacetate
7-C	142.311	12-C	146.455	2-C	151.741
8-C	133.869	11-C	21.760	6-C	129.323
1-C	128.589	10-C	16.876	1-C	121.591
4-C	120.975	8-C	15.603	5-C	105.659
5-C	113.284	36-C	15.499	4-C	63.818
2-C	108.036	1-C	15.387	7-C	24.997
3-C	103.793	13-C	15.320	13-C	23.385
6-C	96.637	3-C	14.511	12-C	22.139
17-C	−12.527	4-C	14.094	11-C	22.046
33-C	−13.724	31-C	13.771	10-C	21.930
19-C	−13.895	5-C	13.444	9-C	21.861
18-C	−13.914	6-C	13.024	8-C	21.102
32-C	−13.918	7-C	12.407	14-C	14.600
31-C	−14.110	2-C	11.635	15-C	3.962
		9-C	11.045		

**Table 7:** Docking scores of the identified compounds from methanol extract of *C. pectinata* leaves.

Compounds	Docking Score (kcal/mol)			
	2Z5X	5I6X	1ERR	1M17
5-Chloro-1-(trimethylsilyl)-1*H*-indole-2,3-dione 3-[O-(trimethyl- silyl)oxime]	–	–	–	–
Cyclopentadecanone oxime	−4.333	−6.537	−7.685	−4.59
*trans*-2-Dodecen-1-ol trifluoroacetate	−3.155	−2.387	−1.857	−2.674
Standard drugs (Phenelzine/Fluoxetine/Vincristine sulfate)	−5.324	−9.07	−3.896	−3.85

**Table 8:** ADME/T properties of the selected compounds in MECP by SwissADME.

Compounds	Lipinski Rules				Lipinski Violations	Veber Rules	
	MW	HBA	HBD	Log P		nRB	TPSA
5-Chloro-1-(trimethylsilyl)-1*H*-indole-2,3-dione 3-[O-(trimethylsilyl)oxime]	340.95	3	0	2.38	0	3	41.90
Cyclopentadecanone oxime	239.40	2	1	3.55	0	0	32.59
*trans*-2-Dodecen-1-ol trifluoroacetate	280.33	5	0	3.96	0	12	26.30

MW, Molecular weight (<500 g/mol); HBA, Hydrogen bond acceptor (<10); HBD, Hydrogen bond donor (<5); Log P, Lipophilicity (≤5); nRB: number of rotatable bond (≤10); TPSA: topological polar surface area (≤140 Å²).

**Table 9:** Toxicological properties of the selected compounds in MECP.

Parameters	Compounds		
	5-Chloro-1-(trimethylsilyl)-1*H*-Indole- 2,3-dione 3-[O-(trimethylsilyl)oxime]	Cyclopentadecanone Oxime	*trans*-2-Dodecen-1-ol Trifluoroacetate
Ames toxicity	NAT	AT	NAT
Carcinogens	NC	NC	C
Acute oral toxicity	III	III	III
Rat acute toxicity	2.6849	2.1203	2.6831

NAT, Non-Ames toxic; AT, Ames toxic; NC, Non-carcinogenic; C, carcinogenic; NR, Non-required. Category-I means (LD_50_ ≤ 50 mg/kg) and Category-III (500 mg/kg > LD_50_ < 5000 mg/kg).

## Supplementary Materials

The following are available online at https://www.mdpi.com/1424-8247/13/9/232/s1, Table S1: Mulliken atomic charges and NBO charges at different atoms in gas phase of 5-chloro-1-(trimethylsilyl)-1H-indole-2,3-dione 3-[O-(trimethylsilyl)-oxime] computed by B3LYP/methods with 6-31G+ (d,p) basis set. Table S2: Mulliken atomic charges and NBO charges at different atoms in gas phase of cyclopentadecanone oxime computed by B3LYP/methods with 6-31G+ (d,p) basis set. Table S3: Mulliken atomic charges and NBO charges at different atoms in gas phase of trans-2-dodecen-1-ol trifluoroacetate computed by B3LYP/methods with 6-31G+ (d,p) basis set. Figure S1: Total ionic chromatogram (TIC) of MECP by GC-MS. Figure S2: Fourier transform Infrared- (FT-IR) spectrum of 5-chloro-1-(trimethylsilyl)-1H-indole-2,3-dione 3-[O-(trimethylsilyl)-oxime] (A); cyclopentadecanone oxime (B); trans-2-dodecen-1-ol trifluoroacetate (C), respectively in the wavenumber range 4000 – 0 cm-1. Figure S3: Calculated 1H-NMR isotropic chemical shift spectrum of 5-chloro-1-(trimethylsilyl)-1H-indole-2,3-dione 3-[O-(trimethylsilyl)-oxime] (A); cyclopentadecanone oxime (B); trans-2-dodecen-1-ol trifluoroacetate (C), respectively in chloroform solvent. Figure S4: Calculated 13C NMR isotropic chemical shift spectrum of 5-chloro-1-(trimethylsilyl)-1H-indole-2,3-dione 3-[O-(trimethylsilyl)-oxime] (A); cyclopentadecanone oxime (B); trans-2-dodecen-1-ol trifluoroacetate (C), respectively in chloroform solvent. Figure S5: 3D and 2D interactions of cyclopentadecanone oxime (A); trans-2-dodecen-1-ol trifluoroacetate (B); phenelzine (C), against the human monoamine oxidase A (PDB: 2Z5X) for antidepressant activity; Figure S6: 3D and 2D interactions of cyclopentadecanone oxime (A); trans-2-dodecen-1-ol trifluoroacetate (B); fluoxetine (C), against the human serotonin receptor (PDB: 5I6X) for antidepressant activity; Figure S7: 3D and 2D interactions of cyclopentadecanone oxime (A); *trans*-2-dodecen-1-ol trifluoroacetate (B); vincristine sulfate (C), against the human estrogen receptor (PDB ID: 1ERR) for cytotoxicity activity; Figure S8: 3D and 2D interactions of cyclopentadecanone oxime (A); trans-2-dodecen-1-ol trifluoroacetate (B); vincristine sulfate (C), against the epidermal growth factor receptor tyrosine kinase (PDB ID: 1M17) for cytotoxicity activity.

## Abbreviations

MECP	methanol extract of *C. pectinata* leaves
GC-MS	Gas Chromatography-Mass Spectroscopy
IP	intraperitoneal
b.w.	body weight
MAO	monoamine oxidase
NMR	nuclear magnetic resonance
DFT	density functional theory
ADME/T	absorption, distribution, metabolism, excretion, and toxicity
PDB	protein data bank
SEM	standard error mean
ANOVA	one-way analysis of variance
FMOs	frontier molecular orbitals
HOMO	highest occupied molecular orbital
LUMO	lowest unoccupied molecular orbital
MESP	molecular electrostatic potential
NBO	natural bond orbital
