# Supplementary material for: Biological Evaluation, DFT Calculations and Molecular Docking Studies on the Antidepressant and Cytotoxicity Activities of Cycas pectinata Buch.-Ham. Compounds
Source: Pharmaceuticals (Basel). 2020 Sep 3;13(9):232. doi: 10.3390/ph13090232 (PMC7557754; doi:10.3390/ph13090232)
Supplement: Supplementary file 1 [file pharmaceuticals-13-00232-s001.docx]

***Supplementary Materials***

**Biological Evaluation, DFT Calculations and Molecular Docking Studies on the Antidepressant and Cytotoxicity Activities of *Cycas pectinata* Buch.-Ham. Compounds**

**Jinnat Rahman ^1,†^, Abu Montakim Tareq ^1,†^, Md. Mohotasin Hossain ^1^, Shahenur Alam Sakib ^2^, Mohammad Nazmul Islam ^1^, Md. Hazrat Ali ^1^, A. B. M. Neshar Uddin ^1^, Muminul Hoque ^1^, Mst. Samima Nasrin ^1^, Talha Bin Emran ^3^, Raffaele Capasso ^4,*^, A. S. M. Ali Reza ^1, 5,*^ and Jesus Simal-Gandara ^6,*^**

^1^ Department of Pharmacy, International Islamic University Chittagong, Kumira, Chittagong-4318, Bangladesh; jinnatrahman14@gmail.com (J.R.); montakim0.abu@gmail.com (A.M.T.); mdjisan16@gmail.com (M.M.H.); sayeadiiuc@gmail.com (M.N.I.); hazratpharm@gmail.com (M.H.A.); nesharuddinemon1@gmail.com (A.B.M.N.U.); muminul359@gmail.com (M.H.); shathy_ru@yahoo.com (M.S.N)

^2^ Department of Theoretical and Computational Chemistry, University of Dhaka, Dhaka-1000, Bangladesh; sakibhasaniiuc@gmail.com (S.A.S.)

^3^ Department of Pharmacy, BGC Trust University Bangladesh, Chittagong-4381, Bangladesh; talhabmb@bgctub.ac.bd (T.B.E.)

^4^ Department of Agricultural Sciences, University of Naples Federico II, 80055 Portici, Italy

^5^ Department of Biochemistry and Molecular Biology, University of Chittagong, Chittagong-4331, Bangladesh

^6^ Nutrition and Bromatology Group, Department of Analytical and Food Chemistry, Faculty of 21 Food Science and Technology, University of Vigo – Ourense Campus, E32004 Ourense, Spain; jsimal@uvigo.es (J.S.G.)

*† These authors contributed equally to this work*

***** Correspondence: jsimal@uvigo.es (J.S.G.); rafcapas@unina.it (R.C.); alirezaru@gmail.com (A.S.M.A.R.)

Tel.: +34-988-387000 (J.S.G.); +39‐081‐678664 (R.C.); +8801722-584336 (A.S.M.A.R.)

**Table S1:** Mulliken atomic charges and NBO charges at different atoms in gas phase of 5-chloro-1-(trimethylsilyl)-1*H*-indole-2,3-dione 3-[O-(trimethylsilyl)-oxime] computed by B3LYP/methods with 6-31G+ (d, p) basis set.

|  | | **Atomic Charges**  **5-chloro-1-(trimethylsilyl)-1*H*-indole-2,3-dione 3-[O-(trimethylsilyl)-oxime]** | |
| --- | --- | --- | --- |
| **Sl. No.** | **Atom** | **Mulliken Charges** | **NBO Charges** |
| 1 | C | 0.235 | 0.187 |
| 2 | C | 0.280 | -0.091 |
| 3 | C | -0.643 | -0.211 |
| 4 | C | 0.458 | -0.041 |
| 5 | C | -0.732 | -0.235 |
| 6 | C | -0.417 | -0.266 |
| 7 | C | -0.219 | 0.620 |
| 8 | C | -0.276 | 0.110 |
| 9 | H | 0.152 | 0.275 |
| 10 | H | 0.146 | 0.265 |
| 11 | H | 0.130 | 0.250 |
| 12 | Cl | 0.417 | -0.046 |
| 13 | N | -0.021 | -0.800 |
| 14 | N | -0.481 | -0.090 |
| 15 | O | -0.258 | -0.539 |
| 16 | Si | **1.710** | 1.849 |
| 17 | C | -0.862 | -1.202 |
| 18 | C | **-0.877** | -1.209 |
| 19 | C | **-0.877** | -1.209 |
| 20 | H | 0.157 | 0.254 |
| 21 | H | 0.196 | 0.279 |
| 22 | H | 0.197 | 0.280 |
| 23 | H | 0.170 | 0.263 |
| 24 | H | 0.185 | 0.269 |
| 25 | H | 0.170 | 0.259 |
| 26 | H | 0.170 | 0.263 |
| 27 | H | 0.170 | 0.259 |
| 28 | H | 0.184 | 0.269 |
| 29 | O | 0.194 | -0.659 |
| 30 | Si | 1.252 | **1.905** |
| 31 | C | -0.810 | **-1.219** |
| 32 | C | -0.833 | **-1.224** |
| 33 | C | -0.828 | **-1.225** |
| 34 | H | 0.179 | 0.273 |
| 35 | H | 0.166 | 0.260 |
| 36 | H | 0.178 | 0.272 |
| 37 | H | 0.175 | 0.268 |
| 38 | H | 0.164 | 0.262 |
| 39 | H | 0.180 | 0.274 |
| 40 | H | 0.180 | 0.273 |
| 41 | H | 0.164 | 0.262 |
| 42 | H | 0.175 | 0.268 |

**Table S2:** Mulliken atomic charges and NBO charges at different atoms in gas phase of cyclopentadecanone oxime computed by B3LYP/methods with 6-31G+ (d, p) basis set.

|  | | **Atomic Charges**  Cyclopentadecanone oxime | |
| --- | --- | --- | --- |
| **Sl. No.** | **Atom** | **Mulliken Charges** | **NBO Charges** |
| 1 | C | -0.307 | -0.462 |
| 2 | C | -0.310 | -0.468 |
| 3 | C | -0.302 | -0.476 |
| 4 | C | -0.311 | -0.471 |
| 5 | C | -0.298 | -0.466 |
| 6 | C | -0.298 | -0.475 |
| 7 | C | -0.293 | -0.463 |
| 8 | C | -0.306 | -0.479 |
| 9 | C | -0.309 | -0.469 |
| 10 | C | -0.355 | -0.525 |
| 11 | C | -0.346 | -0.497 |
| 12 | C | 0.273 | 0.238 |
| 13 | C | -0.290 | -0.466 |
| 14 | N | -0.209 | -0.170 |
| 15 | O | **-0.638** | **-0.620** |
| 16 | H | **0.428** | **0.491** |
| 17 | H | 0.169 | 0.236 |
| 18 | H | 0.214 | 0.278 |
| 19 | H | 0.187 | 0.259 |
| 20 | H | 0.154 | 0.236 |
| 21 | H | 0.161 | 0.244 |
| 22 | H | 0.152 | 0.240 |
| 23 | H | 0.151 | 0.239 |
| 24 | H | 0.151 | 0.237 |
| 25 | H | 0.150 | 0.236 |
| 26 | H | 0.150 | 0.238 |
| 27 | H | 0.155 | 0.241 |
| 28 | H | 0.151 | 0.218 |
| 29 | H | 0.173 | 0.250 |
| 30 | H | 0.154 | 0.239 |
| 31 | C | -0.298 | -0.472 |
| 32 | H | 0.151 | 0.235 |
| 33 | H | 0.155 | 0.237 |
| 34 | H | 0.148 | 0.224 |
| 35 | H | 0.153 | 0.244 |
| 36 | C | -0.303 | -0.479 |
| 37 | H | 0.151 | 0.238 |
| 38 | H | 0.151 | 0.238 |
| 39 | H | 0.192 | 0.267 |
| 40 | H | 0.172 | 0.234 |
| 41 | H | 0.156 | 0.241 |
| 42 | H | 0.152 | 0.240 |
| 43 | H | 0.158 | 0.226 |
| 44 | H | 0.155 | 0.244 |
| 45 | H | 0.156 | 0.246 |
| 46 | H | 0.150 | 0.221 |

**Table S3:** Mulliken atomic charges and NBO charges at different atoms in gas phase of *trans*-2-dodecen-1-ol trifluoroacetate computed by B3LYP/methods with 6-31G+ (d, p) basis set.

|  | | **Atomic charges**  ***trans*-2-dodecen-1-ol trifluoroacetate** | |
| --- | --- | --- | --- |
| **No** | **Atom** | **Mulliken charges** | **NBO charges** |
| 1 | C | **0.769** | **0.983** |
| 2 | C | 0.467 | 0.675 |
| 3 | O | **-0.480** | -0.519 |
| 4 | C | -0.105 | -0.163 |
| 5 | C | -0.077 | -0.268 |
| 6 | C | -0.066 | -0.169 |
| 7 | C | -0.306 | -0.497 |
| 8 | C | -0.240 | -0.459 |
| 9 | C | -0.239 | -0.465 |
| 10 | C | -0.240 | -0.463 |
| 11 | C | -0.239 | -0.463 |
| 12 | C | -0.239 | -0.464 |
| 13 | C | -0.231 | -0.466 |
| 14 | C | -0.237 | -0.466 |
| 15 | C | -0.405 | -0.697 |
| 16 | H | 0.130 | 0.239 |
| 17 | H | 0.131 | 0.232 |
| 18 | H | 0.131 | 0.232 |
| 19 | H | 0.123 | 0.233 |
| 20 | H | 0.123 | 0.233 |
| 21 | H | 0.119 | 0.231 |
| 22 | H | 0.119 | 0.231 |
| 23 | H | 0.120 | 0.232 |
| 24 | H | 0.120 | 0.232 |
| 25 | H | 0.121 | 0.232 |
| 26 | H | 0.121 | 0.232 |
| 27 | H | 0.121 | 0.232 |
| 28 | H | 0.122 | 0.233 |
| 29 | H | 0.123 | 0.233 |
| 30 | H | 0.124 | 0.234 |
| 31 | H | 0.126 | 0.234 |
| 32 | H | 0.131 | 0.239 |
| 33 | H | 0.146 | 0.253 |
| 34 | H | 0.138 | 0.244 |
| 35 | H | 0.127 | 0.228 |
| 36 | H | 0.143 | 0.241 |
| 37 | H | 0.181 | 0.247 |
| 38 | H | 0.184 | 0.252 |
| 39 | F | -0.262 | -0.337 |
| 40 | F | -0.261 | -0.336 |
| 41 | F | -0.258 | -0.331 |
| 42 | O | -0.377 | **-0.524** |


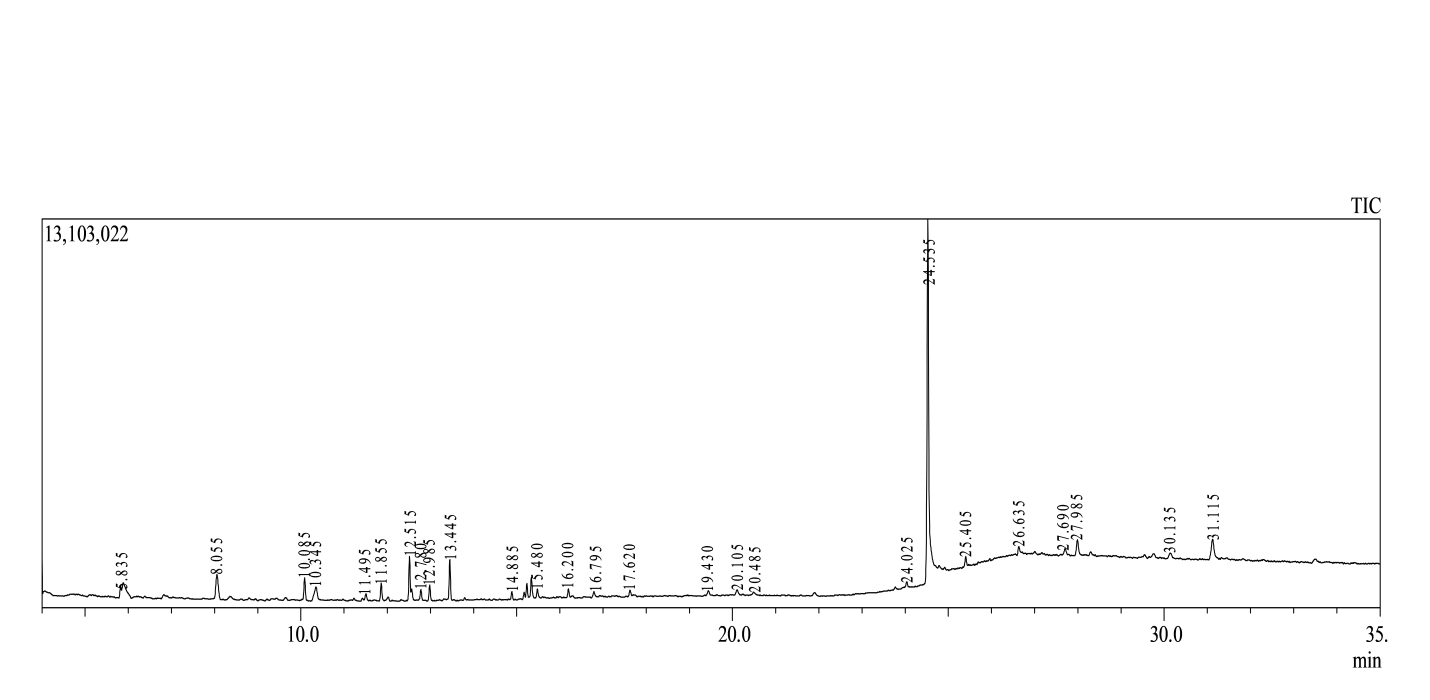


Figure S1: Total ionic chromatogram (TIC) of MECP by GC-MS.

|  | |
| --- | --- |
| 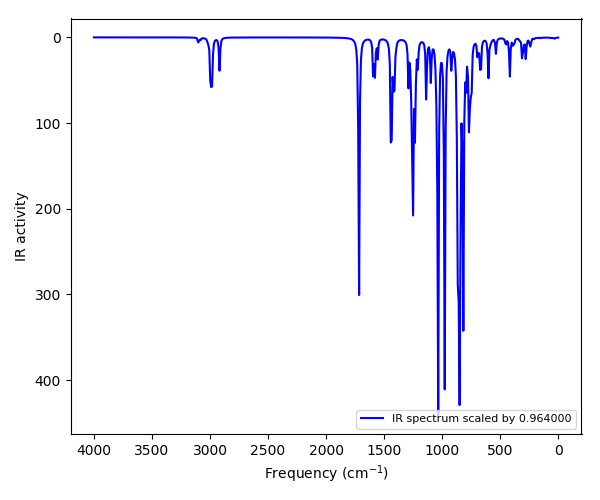  **A** |  |
| 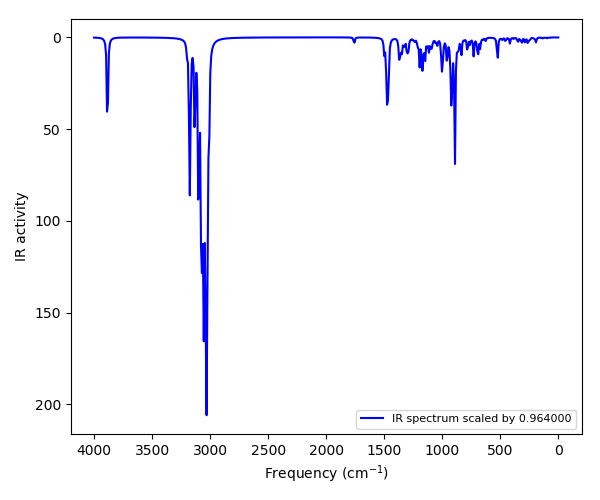  **B** |  |
| 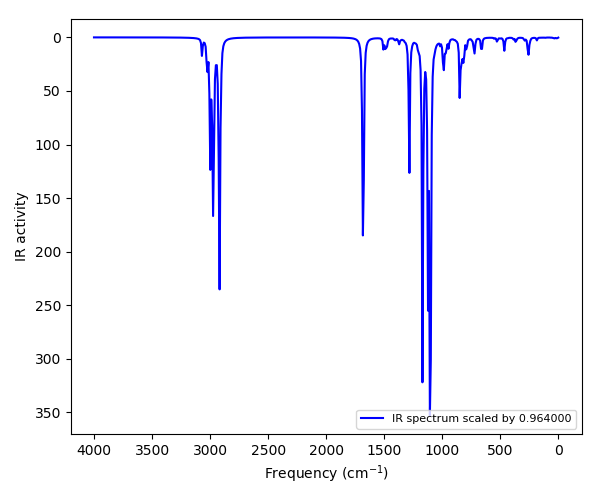  **C** |  |

Figure S2: Fourier transform Infrared- (FT-IR) spectrum of 5-chloro-1-(trimethylsilyl)-1*H*-indole-2,3-dione 3-[O-(trimethylsilyl)-oxime] (A); cyclopentadecanone oxime (B); *trans*-2-dodecen-1-ol trifluoroacetate (C), respectively in the wavenumber range 4000 – 0 cm^-1^.

| 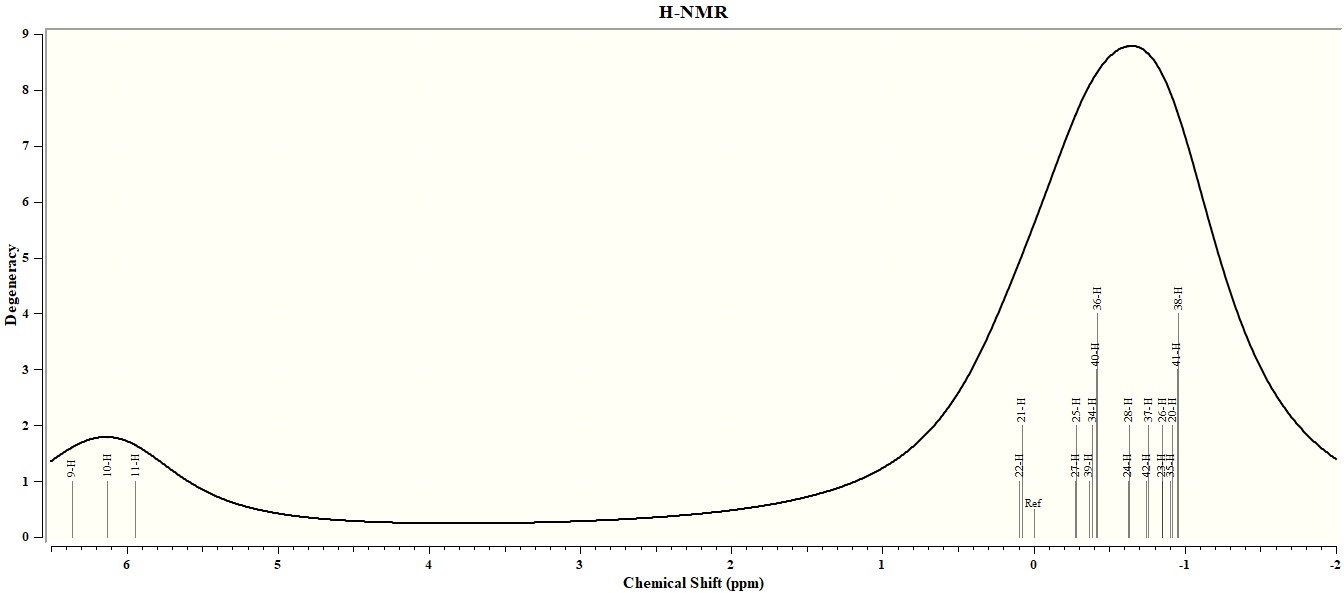  **A** |
| --- |
| 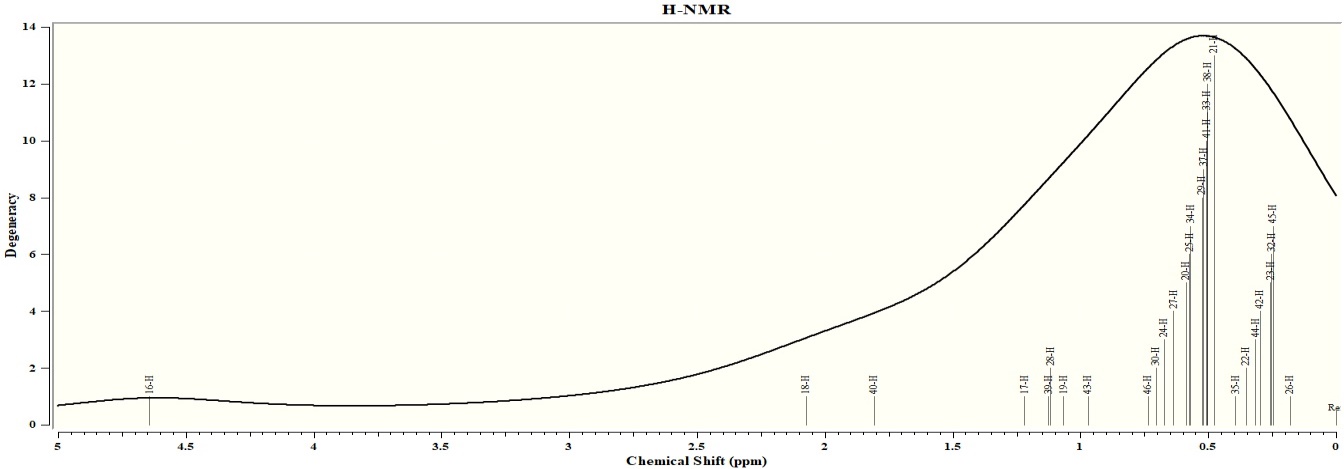  **B** |
| 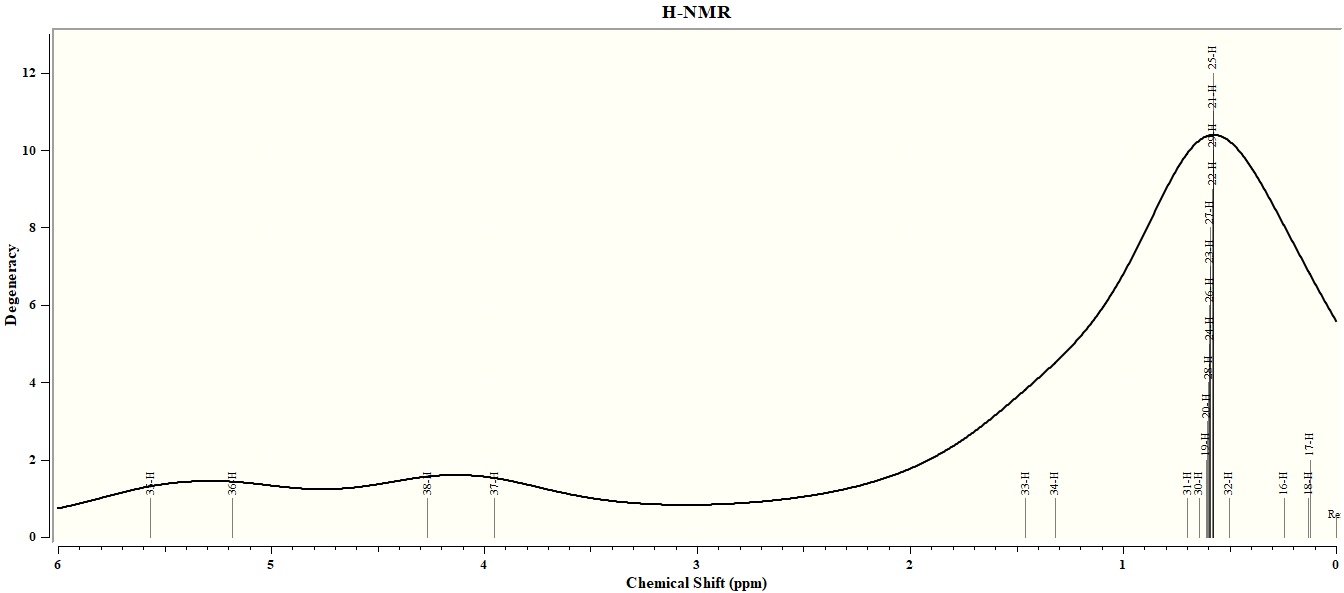  **C** |

Figure S3: Calculated ^1^H NMR isotropic chemical shift spectrum of 5-chloro-1-(trimethylsilyl)-1*H*-indole-2,3-dione 3-[O-(trimethylsilyl)-oxime] (A); cyclopentadecanone oxime (B); *trans*-2-dodecen-1-ol trifluoroacetate (C), respectively in chloroform solvent.

|  | |
| --- | --- |
| 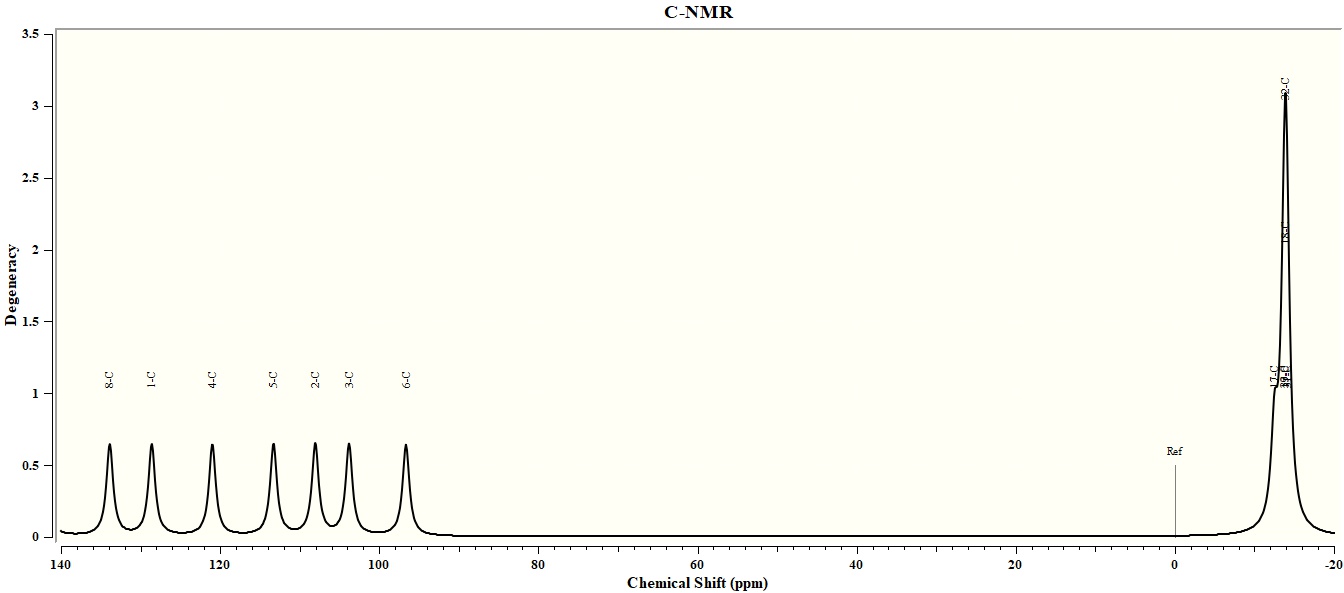  **A** |  |
| 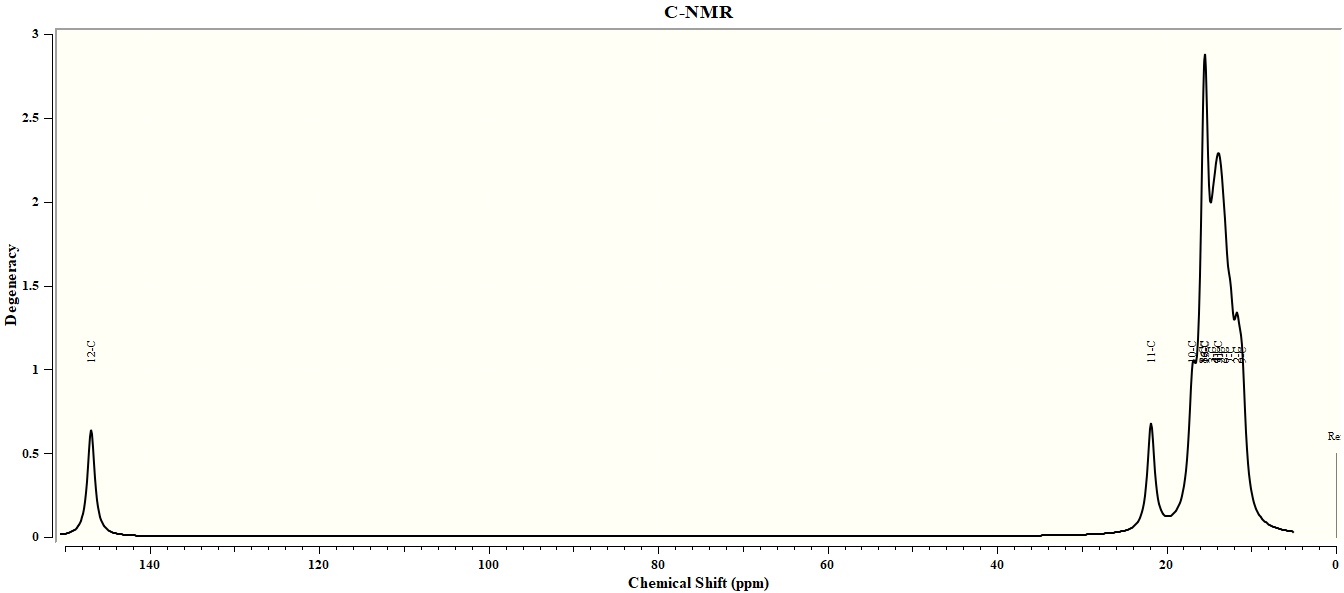  **B** |  |
| 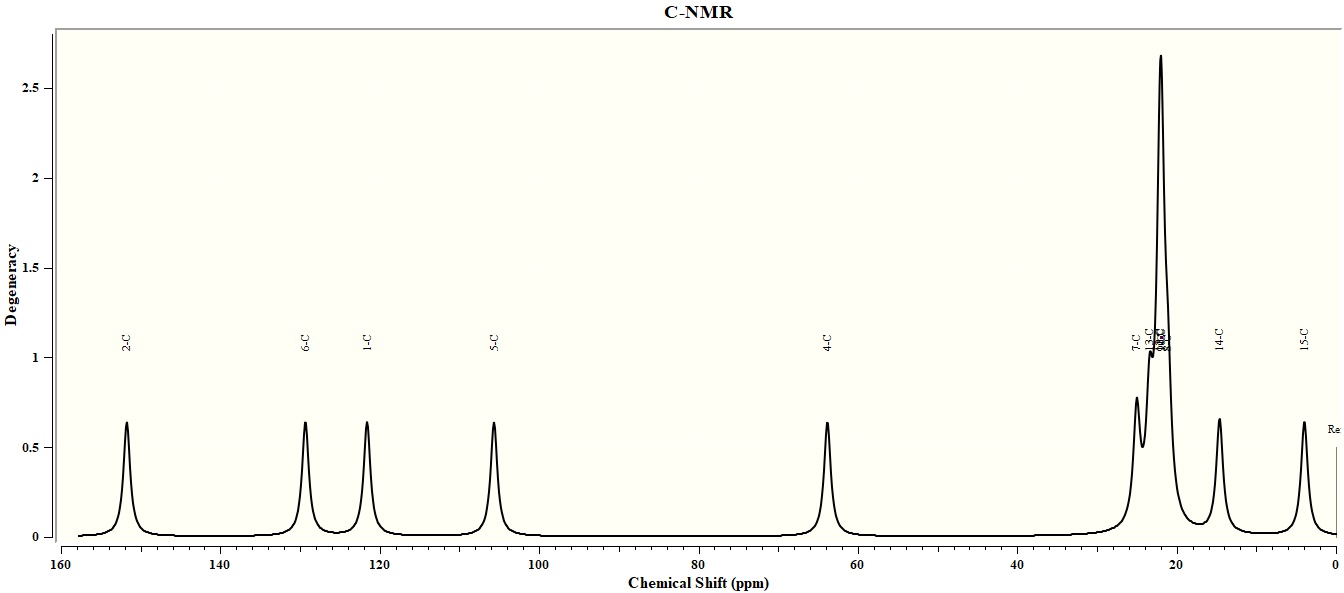  **C** |  |

**Figure S4:** Calculated ^13^C NMR isotropic chemical shift spectrum of 5-chloro-1-(trimethylsilyl)-1*H*-indole-2,3-dione 3-[O-(trimethylsilyl)-oxime] (A); cyclopentadecanone oxime (B); *trans*-2-dodecen-1-ol trifluoroacetate (C), respectively in chloroform solvent.

| 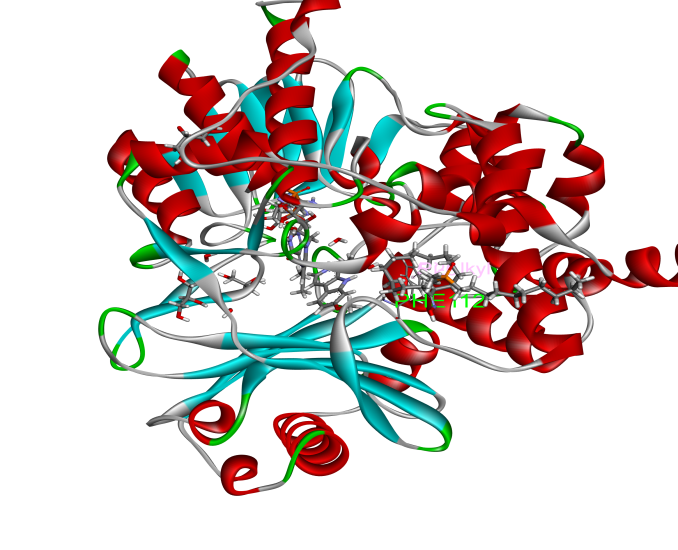  **A** | 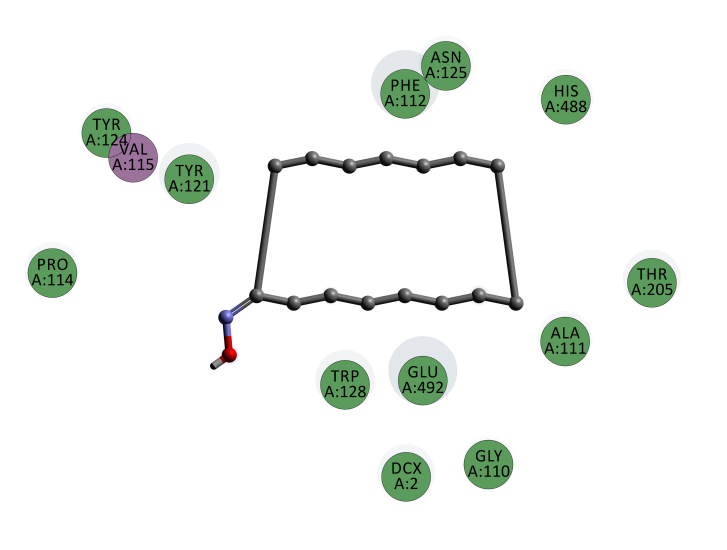 |
| --- | --- |
|  |  |
| 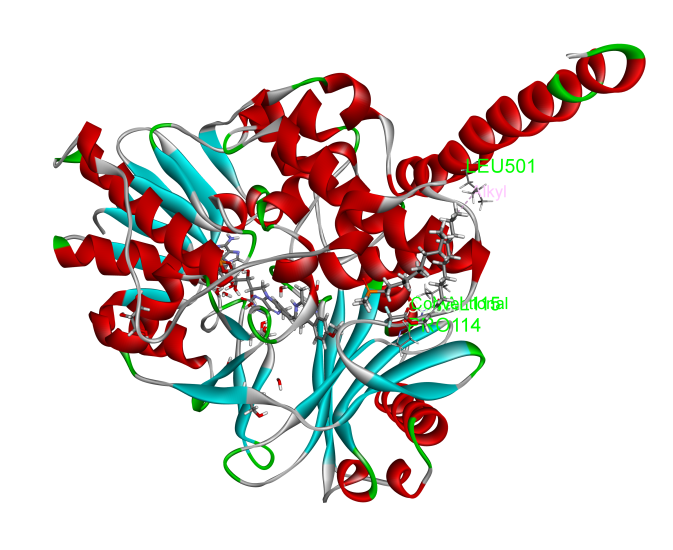  **B** | 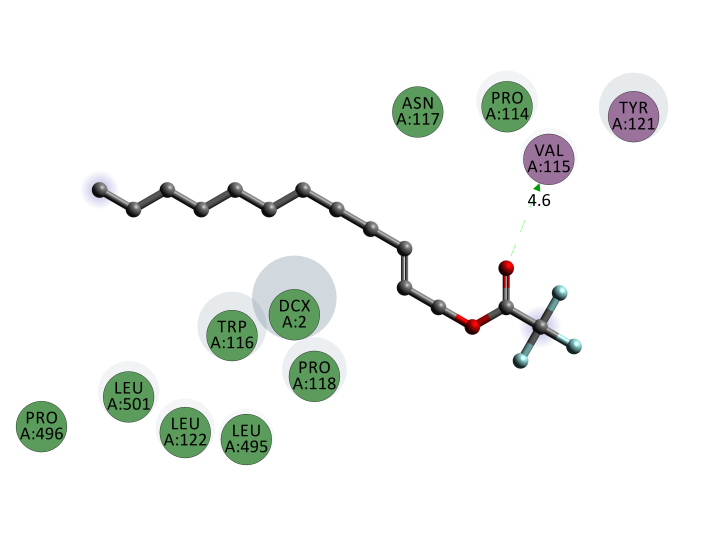 |
| 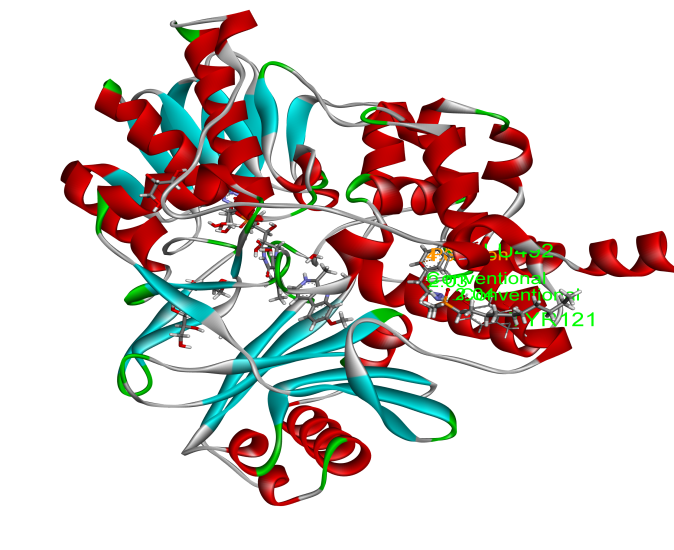  **C** | 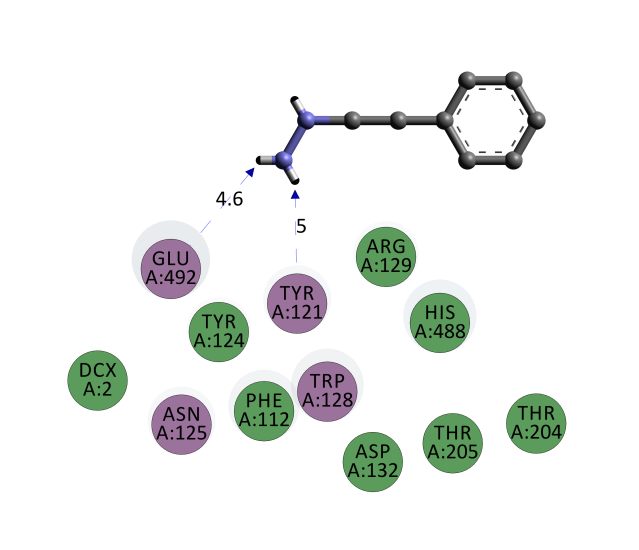 |

Figure S5: 3D and 2D interactions of cyclopentadecanone oxime (A); *trans*-2-dodecen-1-ol trifluoroacetate (B); phenelzine (C), against the human monoamine oxidase A (PDB: 2Z5X) for antidepressant activity.

| 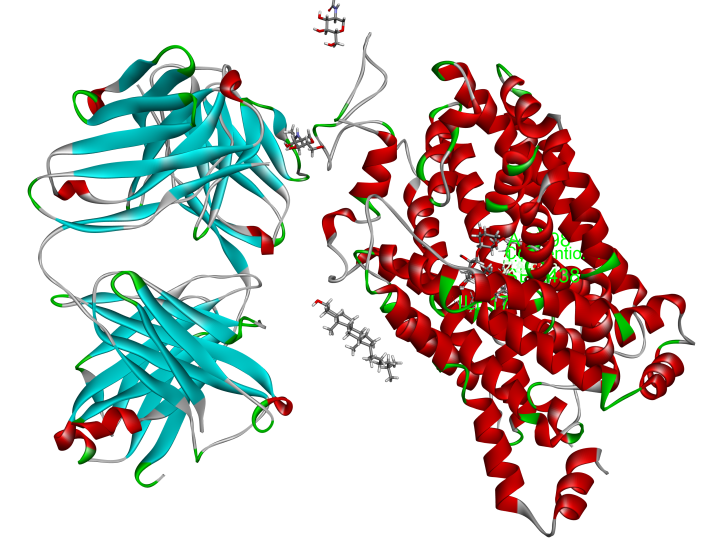  **A** | 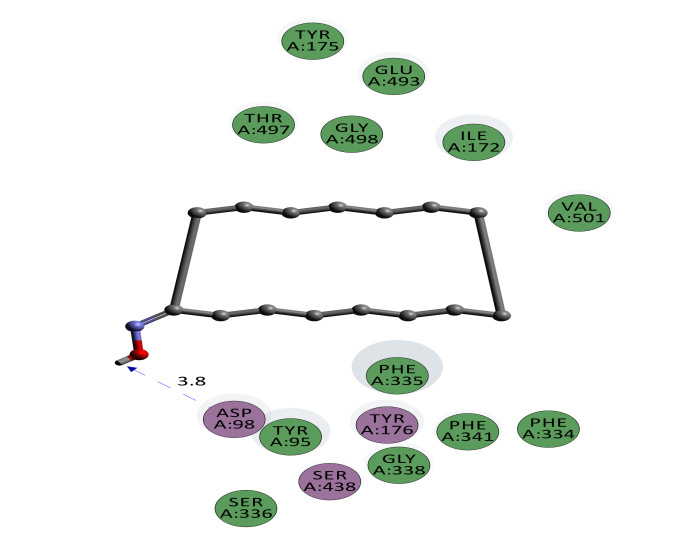 |
| --- | --- |
|  |  |
| 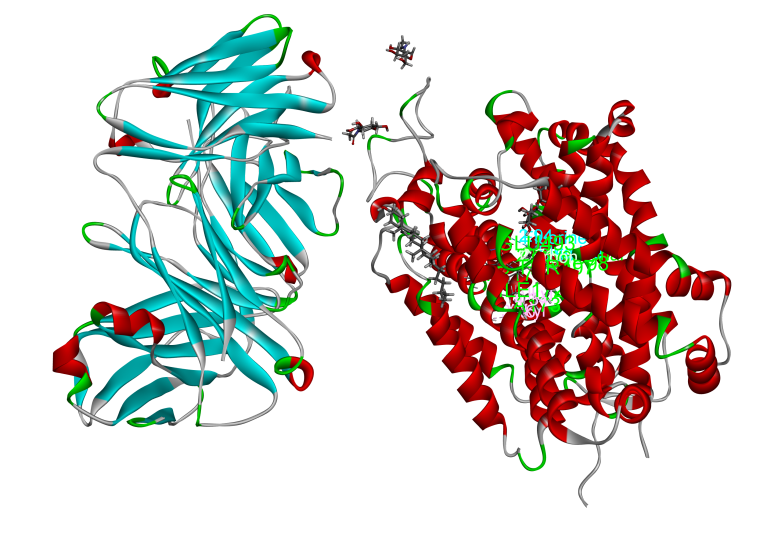  **B** | 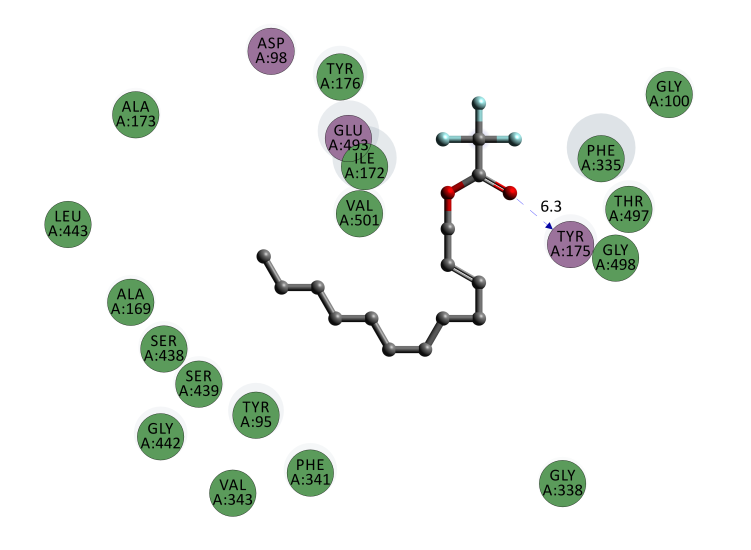 |
| 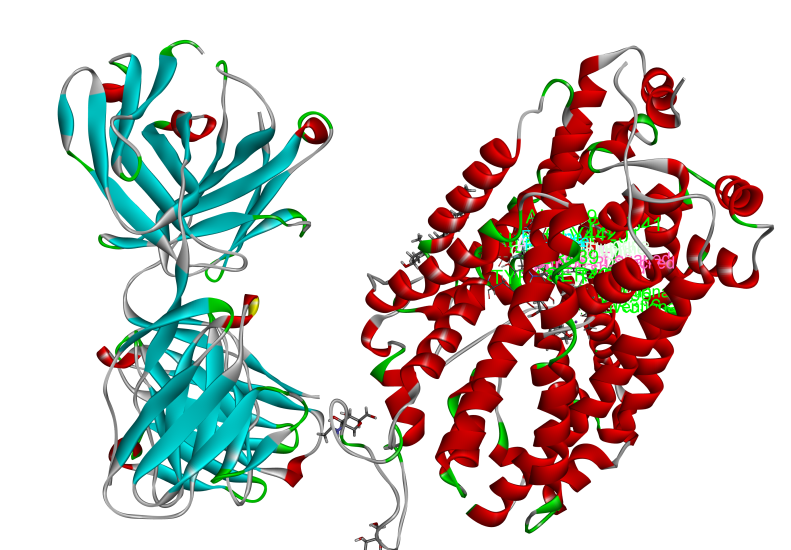  **C** | 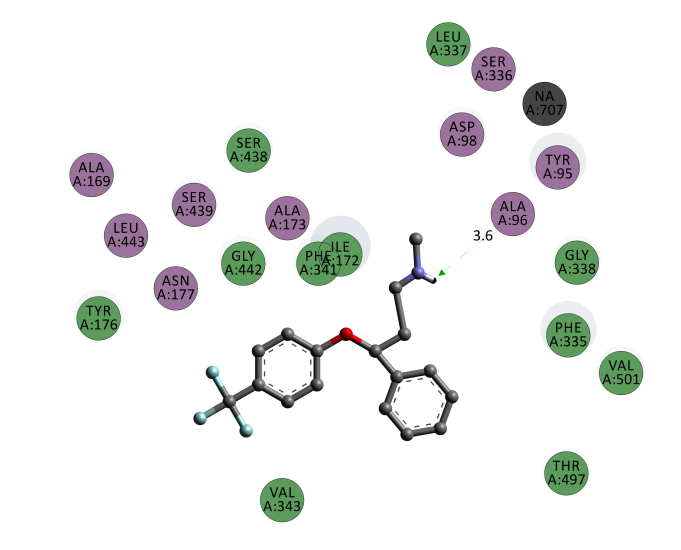 |

Figure S6: 3D and 2D interactions of cyclopentadecanone oxime (A); *trans*-2-dodecen-1-ol trifluoroacetate (B); fluoxetine (C), against the human serotonin receptor (PDB: 5I6X) for antidepressant activity.

| 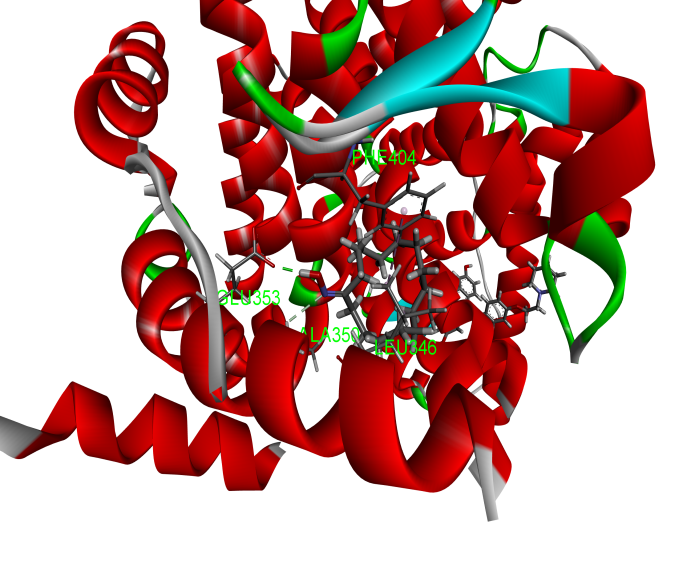  **A** | 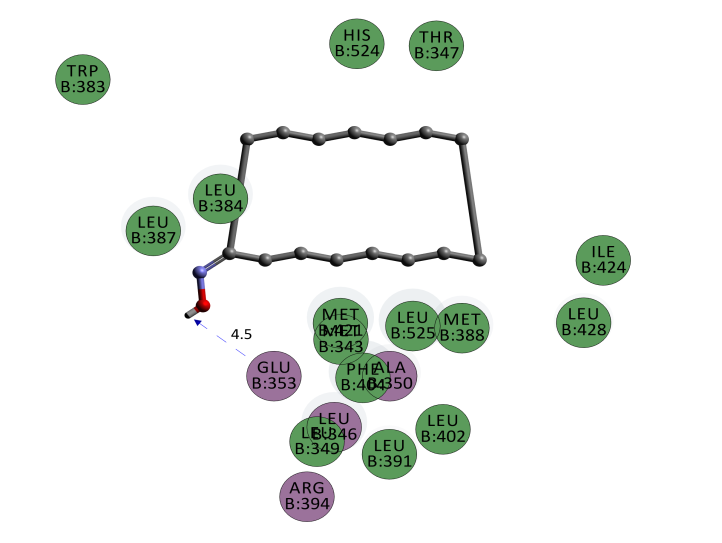 |
| --- | --- |
|  |  |
| 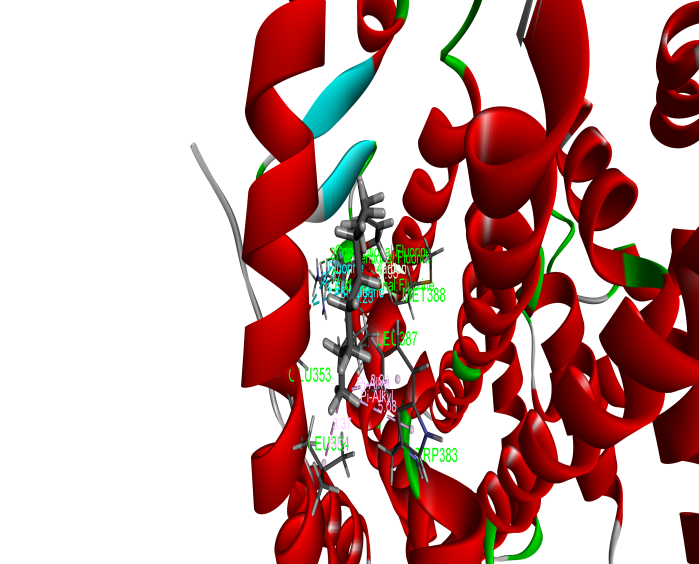  **B** | 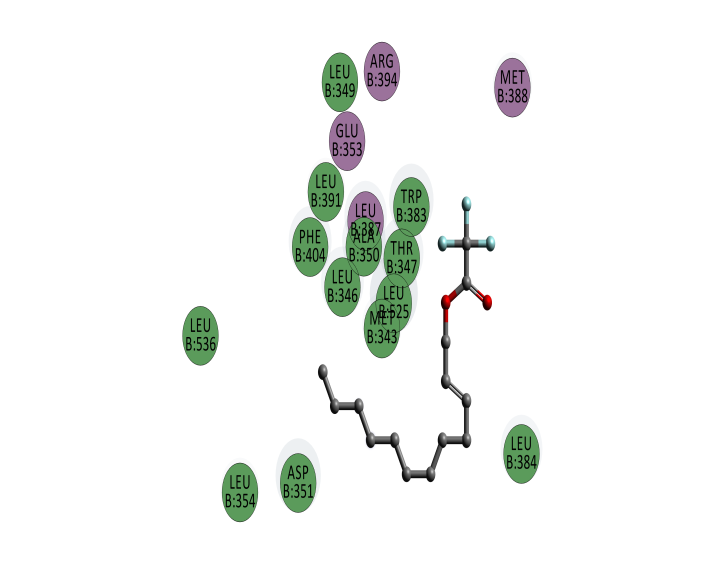 |
| 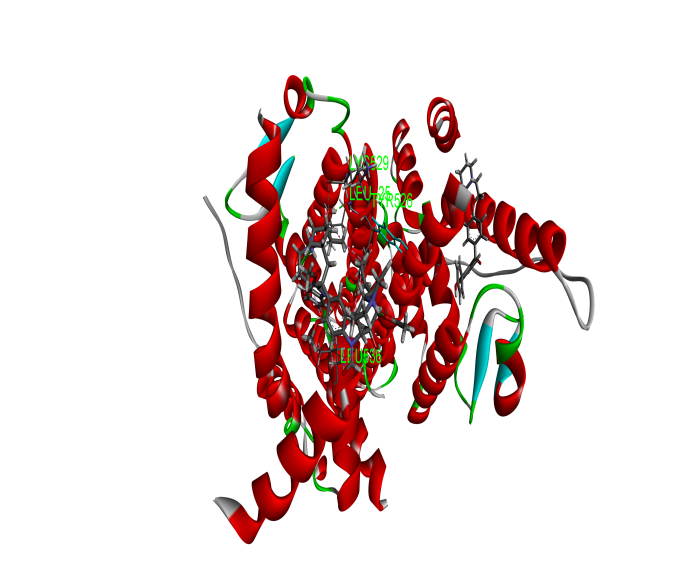  **C** | 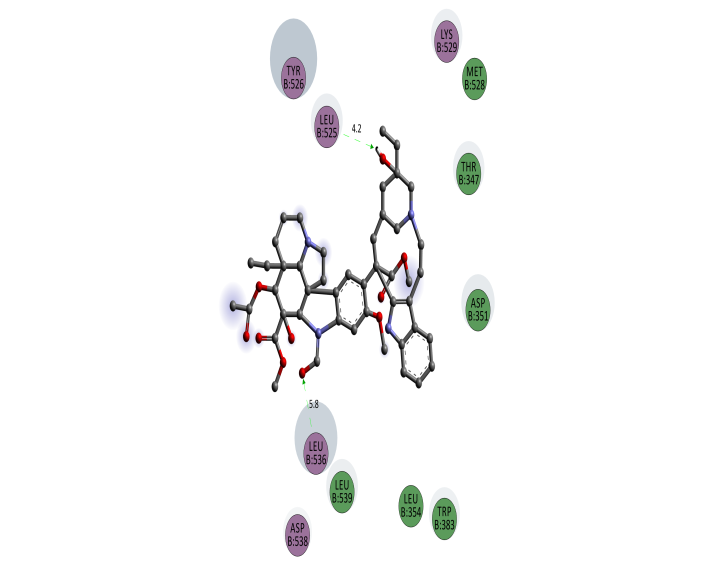 |

Figure S7: 3D and 2D interactions of cyclopentadecanone oxime (A); *trans*-2-dodecen-1-ol trifluoroacetate (B); vincristine sulfate (C), against the human estrogen receptor (PDB ID: 1ERR) for cytotoxicity activity.

| 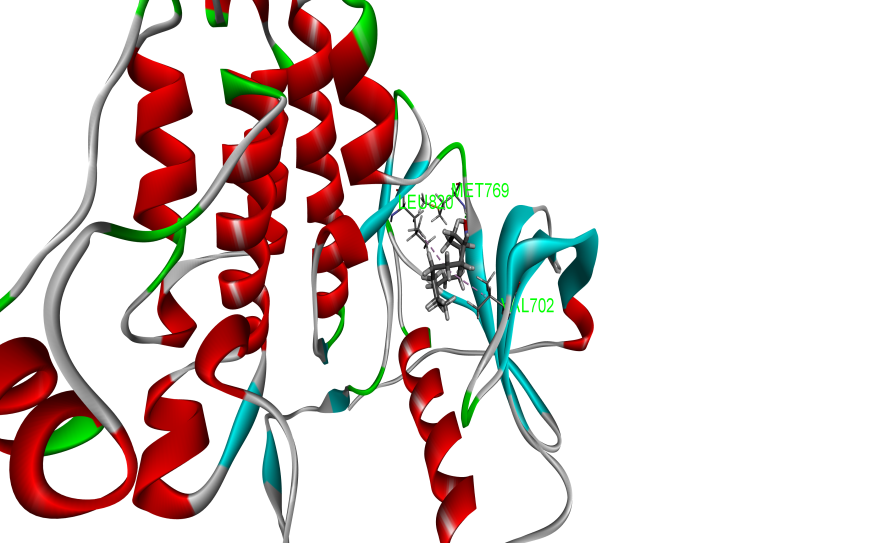  **A** | 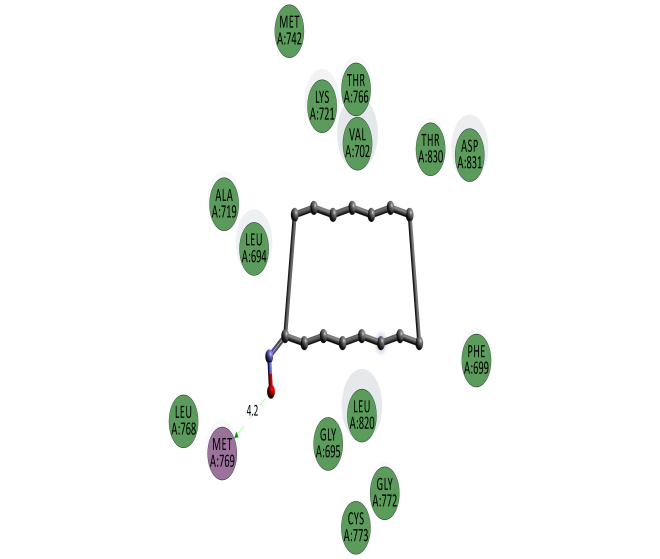 |
| --- | --- |
|  |  |
| 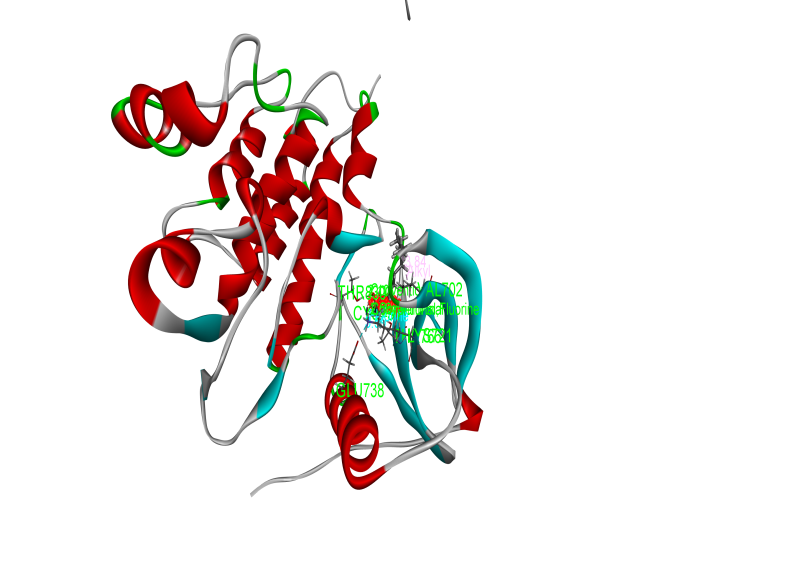  **B** | 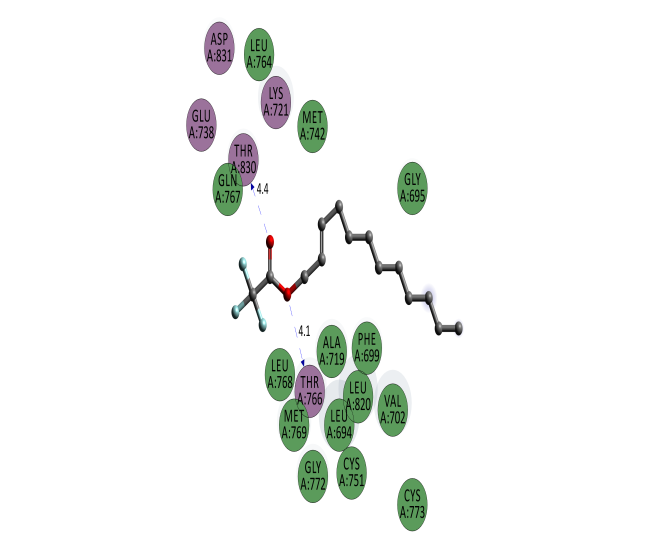 |
| 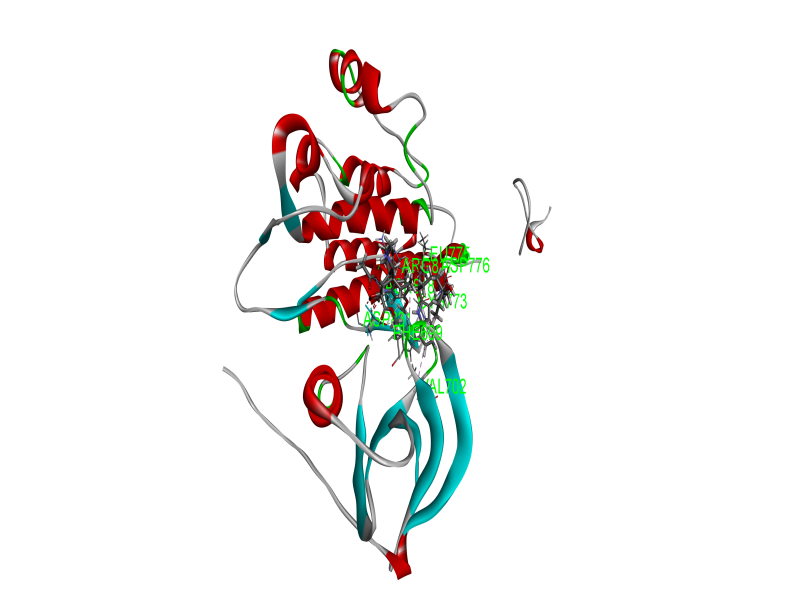  **C** | 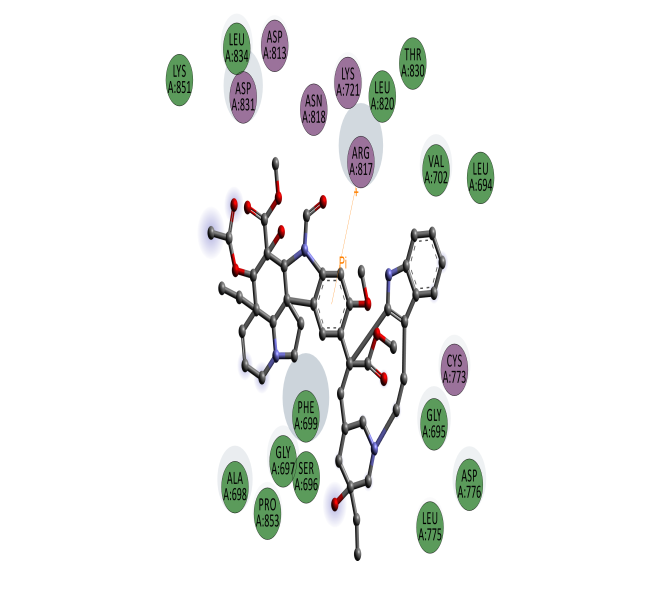 |

Figure S8: 3D and 2D interactions of cyclopentadecanone oxime (A); *trans*-2-dodecen-1-ol trifluoroacetate (B); vincristine sulfate (C), against the epidermal growth factor receptor tyrosine kinase (PDB ID: 1M17) for cytotoxicity activity.
